# Transition metal redox switches for reversible “on/off” and “slow/fast” single-molecule magnet behaviour in dysprosium and erbium bis-diamidoferrocene complexes†
†Electronic supplementary information (ESI) available: Additional figures and tables. CCDC 1566080 and 1566081. For ESI and crystallographic data in CIF or other electronic format see DOI: 10.1039/c7sc03380j


**DOI:** 10.1039/c7sc03380j

**Published:** 2017-10-02

**Authors:** Courtney M. Dickie, Alexander L. Laughlin, Joshua D. Wofford, Nattamai S. Bhuvanesh, Michael Nippe

**Affiliations:** a Department of Chemistry , Texas A&M University , 3255 TAMU , College Station , TX , 77843 USA . Email: nippe@chem.tamu.edu; b Department of Chemistry and Biochemistry , University of California , 607 Charles E. Young Drive East , Los Angeles , California 90095 , USA

## Abstract

Single-molecule magnets (SMMs) are considered viable candidates for next-generation data storage and quantum computing. Systems featuring switchability of their magnetization dynamics are particularly interesting with respect to accessing more complex logic gates and device architectures. Here we show that transition metal based redox events can be exploited to enable reversible switchability of slow magnetic relaxation of magnetically anisotropic lanthanide ions. Specifically, we report anionic homoleptic bis-diamidoferrocene complexes of Dy^3+^ (oblate) and Er^3+^ (prolate) which can be reversibly oxidized by one electron to yield their respective charge neutral redox partners (Dy: **[1]^–^**, **1**; Er: **[2]^–^**, **2**). Importantly, compounds **1** and **2** are thermally stable which allowed for detailed studies of their magnetization dynamics. We show that the Dy^3+^**[1]^–^**/**1** system can function as an “on”/“off” or a “slow”/“fast” redox switchable SMM system in the absence or presence of applied dc fields, respectively. The Er^3+^ based **[2]^–^**/**2** system features “on”/“off” switchability of SMM properties in the presence of applied fields. Results from electrochemical investigations, UV-vis-NIR spectroscopy, and ^57^Fe Mössbauer spectroscopy indicate the presence of significant electronic communication between the mixed-valent Fe ions in **1** and **2** in both solution and solid state. This comparative evaluation of redox-switchable magnetization dynamics in low coordinate lanthanide complexes may be used as a potential blueprint toward the development of future switchable magnetic materials.

## Abstract 2

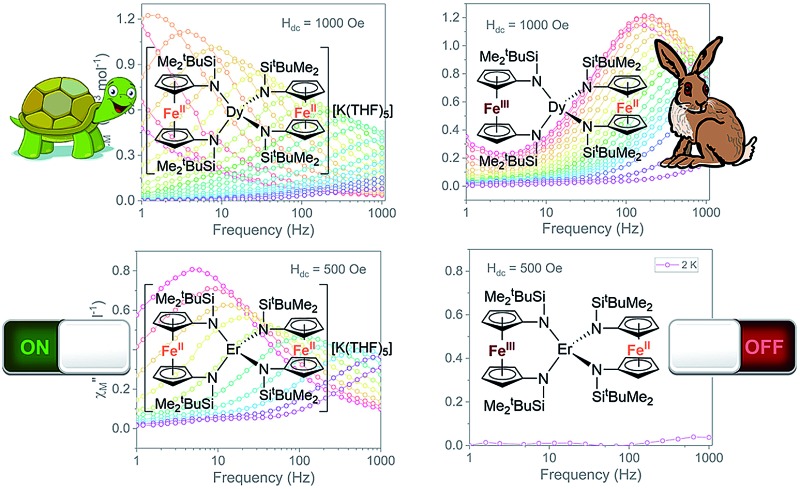
We present an in-depth experimental study of a new class of heterometallic, redox-switchable single-molecule magnets (SMMs).

## Introduction

Single-molecule magnets (SMMs) have been attracting considerable attention for potential applications in quantum computing, high-density data storage and molecular spintronics.^1^–^5^ This fascinating class of compounds is defined by a bistable magnetic ground state and an energy barrier (*U*_eff_) to reorientation of their molecular spin.^6^–^8^ The height of this barrier is determined by the magnetic anisotropy of the system and tunable *via* ligand field considerations. While initial efforts in the field focused on slow magnetic relaxation in multi-nuclear transition metal complexes,^9^,^10^ complementary efforts have led to the development of lanthanide-based single-ion magnets (SIMs).^11^,^12^ Lanthanide ions are attractive candidates for SIM applications, due to their large inherent magnetic single-ion anisotropy, leading to the development of SIMs with record barriers to spin reorientation, *U*_eff_.^13^–^16^

With the ultimate-goal of utilizing SMMs in devices, the development of methodologies that will allow for the control of dynamic magnetic properties reversibly *via* external stimuli is an essential aspect. Possible stimuli include light, temperature, pressure, chemical, dc field, and electric potential.^17^–^22^ Redox-active SMMs present an exciting route for the reversible modulation of magnetic properties using an electric potential.^23^–^30^ Notably, the first reported class of lanthanide ion-based SIMs, the Tb^3+^ phthalocyanine double-decker complexes, are redox-switchable SIMs, undergoing changes in magnetization dynamics and hysteretic behaviour upon electrochemical generation of an open shell π-system.^12^,^31^,^32^

There have been several reported examples of redox-switchability in multinuclear transition metal and lanthanide based SMMs. In these systems, the oxidation and/or reduction of either a ligand^23^,^33^–^35^ or a metal centre^24^–^26^,^36^ facilitates magnetic exchange interactions; turning the SMM properties “on” or “off”. In addition to these “on”/“off” examples, in some cases adding and/or removing an electron has been shown to improve SMM properties.^27^–^30^

Aside from the Tb^3+^ and Dy^3+^ double-decker lanthanide(iii) phthalocyanine complexes,^12^,^37^ to our knowledge there is only one previously reported example of proven redox controllable dynamic magnetic properties in a mononuclear lanthanide(iii)-based SIM. In that example an intramolecularly attached Ru^2/3+^ redox switch was utilized to modify the magnetic relaxation dynamics of a Dy^3+^-based SIM.^29^ The oxidation of Ru^2+^ to Ru^3+^ was found to enhance slow magnetic relaxation, either through perturbations of the ligand field or the addition of another spin-carrier to the system. However, this interesting system suffered from limited thermal stability at room temperature.

The reversible redox properties of ferrocene, FeCp_2_, make it an attractive moiety to include in complexes for redox switchability applications.^38^ This inspired us to evaluate the possibility of utilizing the redox properties of ferrocene-containing ligands to modulate the dynamic magnetic properties of a nearby lanthanide(iii) ion. Previously, Diaconescu *et al.* reported a homoleptic uranium(iv) compound stabilized by two bidentate diamidoferrocene ligands.^39^,^40^ Notably, one-electron oxidation of the complex resulted in a mixed-valent species, indicating strong uranium-mediated electronic communication between the two iron sites. Interestingly, there have been no similar studies reported with lanthanide(iii) bis-diamidoferrocene compounds.

Herein, we present the first class of Ln^3+^ ion-based redox switchable SMMs using the redox chemistry of ferrocene/ferrocenium in the ligand scaffold.^41^–^46^ We show how the reversible one-electron oxidation of Fe^2+^ to Fe^3+^ in Dy^3+^ (oblate electron density) and Er^3+^ (prolate electron density) bis-diaminoferrocene compounds modulates dynamic magnetic properties. Depending on the experimental conditions, these materials can exhibit switchability of their slow magnetic relaxation either between “on” and “off” or between “slow” and “fast”. Remarkably, this is the first example of redox switchable SMM properties observed in an Er^3+^ compound. Additionally, this is the first magnetic investigation of homoleptic, four-coordinate Dy^3+^ and Er^3+^ complexes. This set of compounds has been characterized using X-ray crystallography, dc/ac magnetometry, ^57^Fe Mössbauer spectroscopy, UV-vis-NIR spectroscopy and cyclic voltammetry.

Notably, this is the first example of using the redox chemistry of a transition metal to alter the magnetization dynamics of a lanthanide ion, while maintaining thermal stability of all redox partners. This molecular level study is intended to provide design guidelines for future switchable solid materials.

## Results and discussion

### Synthesis and structural characterisation

**Scheme 1:**
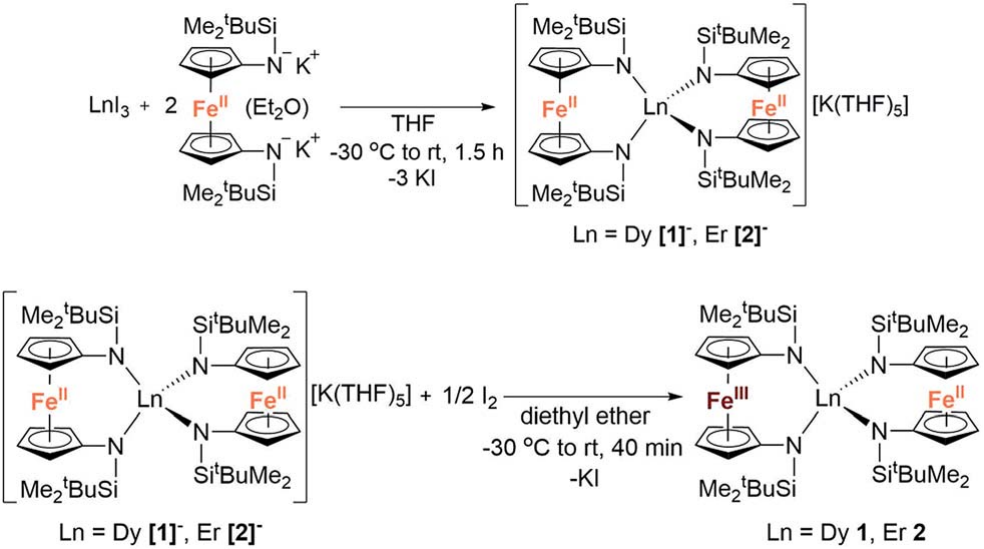
Synthesis of K(thf)_5_[Ln(fc(NSi(*t*-Bu)Me_2_)_2_)_2_] (Ln = Dy **[1]^–^**, Er **[2]^–^**) and Ln(fc[NSi(*t*-Bu)Me_2_]_2_)_2_ (Ln = Dy **1**, Er **2**).

The reaction between LnI_3_ (Ln = Dy or Er) and two equivalents of [K_2_(OEt_2_)]fc[NSi(*t*-Bu)Me_2_]_2_ in thf yielded the anionic lanthanide(iii) species K(thf)_5_[Ln(fc[NSi(*t*-Bu)Me_2_]_2_)_2_] (Dy = **[1]^–^**, Er = **[2]^–^**) (Scheme 1). Yellow plate crystals were grown from a concentrated thf solution layered with hexanes at –30 °C over 24 hours. One-electron oxidation of K(thf)_5_[Ln(fc[NSi(*t*-Bu)Me_2_]_2_)_2_] with half an equivalent of iodine afforded the neutral mixed valent Fe^3+^/Fe^2+^ compounds Dy(fc[NSi(*t*-Bu)Me_2_]_2_)_2_**1** and Er(fc[NSi(*t*-Bu)Me_2_]_2_)_2_**2** (Scheme 1). Dark purple (**1**) and dark red-brown (**2**) block crystals were grown from concentrated hexanes solutions at –30 °C over 24 h.

**Fig. 1:**
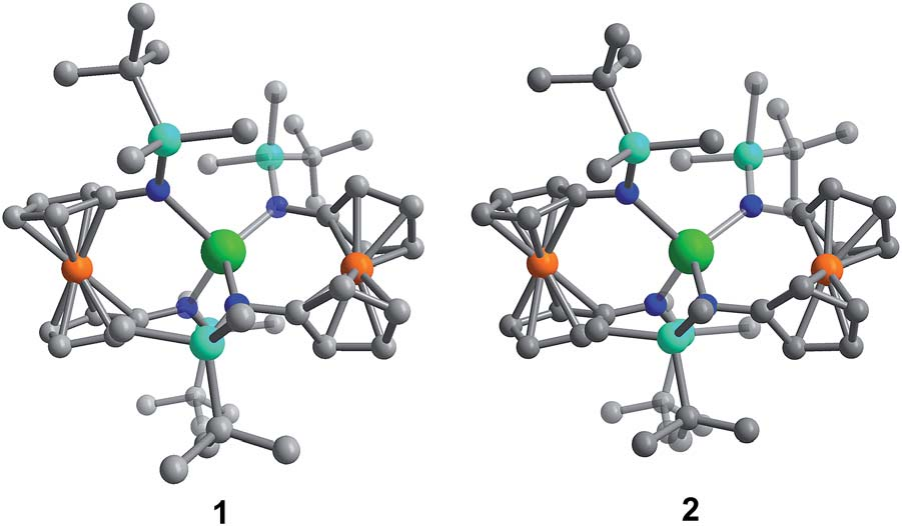
Molecular structure of Dy(fc[NSi(*t*-Bu)Me_2_]_2_)_2_**1** (left) and Er(fc[NSi(*t*-Bu)Me_2_]_2_)_2_**2** (right). Green = Ln, orange = Fe, cyan = Si, blue = N, grey = C. Hydrogen atoms omitted for clarity.

**Fig. 2:**
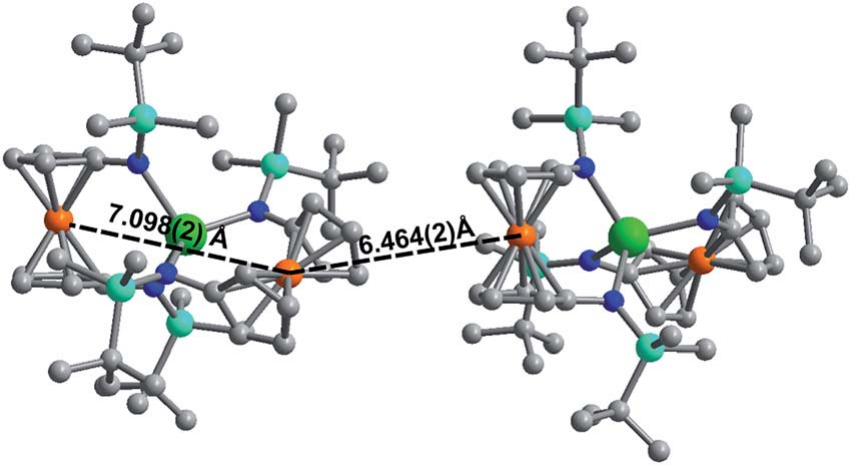
Solid state intra- and intermolecular Fe···Fe distances in **1** at 110 K. Hydrogen atoms omitted for clarity.

Although **[1]^–^** and **[2]^–^** readily crystallize under the experimental conditions, single crystal X-ray diffraction data indicated severe disorder. However, the connectivity of **[1]^–^** and **[2]^–^** could be established and equivalent distances between the Ln^3+^ ion and the two Fe^2+^ ions were found. The neutral, mixed-valent compounds **1** and **2** are isostructural to each other and crystallize in the orthorhombic space group *Pbca* (Fig. 1, Tables S1 and S2†). The central Ln^3+^ ion is four-coordinate, ligated through the nitrogen atoms of the amide moieties of two bidentate diamidoferrocene ligands. Notably, the one-electron oxidized complexes feature lower symmetry around the Ln^3+^ ion. The coordination geometry around the Ln^3+^ ion is distorted tetrahedral, with N-Ln–N angles varying from 100.3(2)° to 131.9(2)° and 102.2(6)° to 126.7(6)° for **1** and **2**, respectively. The differences in Fe–C bond lengths of the two crystallographically independent Fe sites indicate valence localization in the solid state at this temperature (110 K). The two Ln···Fe distances in **1** and **2** are inequivalent, with long Ln···Fe distances of 3.792(2) Å and 3.819(5) Å and short Ln···Fe distances of 3.368(2) Å and 3.498(4) Å (Fig. S1†). The Ln–N distances in **1** and **2** are also inequivalent with longer Ln–N distances (2.370(4), 2.338(4) Å and 2.29(1), 2.27(2) Å) and shorter distances (2.262(4), 2.252(4) Å and 2.22(2), 2.21(2) Å). In the solid state, the closest Fe···Fe distances are intermolecular with separations of 6.464(2) Å and 6.478(5) Å, while the intramolecular Fe···Fe distances are 7.098(2) Å and 7.267(5) Å for **1** and **2**, respectively (Fig. 2 and S2†).

### Magnetic properties

#### Static-field magnetic properties

**Fig. 3:**
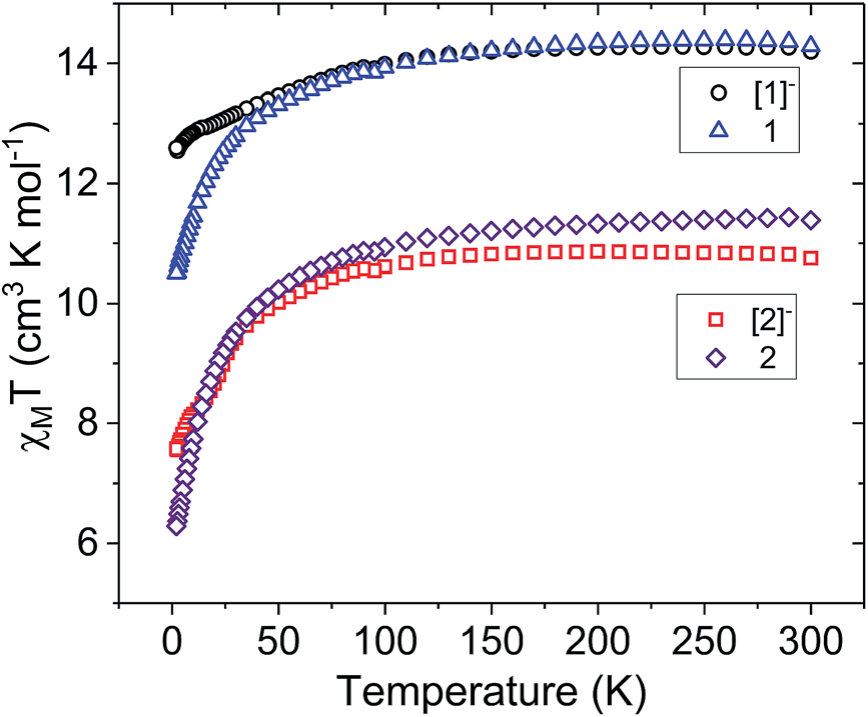
Temperature dependence of the molar magnetic susceptibility times temperature product (*χ*_M_*T*) for compounds **[1]^–^** (black circles), **1** (blue triangles), **[2]^–^** (red squares), and **2** (purple diamonds) under a 1000 Oe dc field.

To investigate static magnetic properties, direct current (dc) magnetic measurements were carried out using crushed polycrystalline samples under an applied field of 1000 Oe from 300 K to 2 K (Fig. 3). The room temperature *χ*_M_*T* value of 14.22 cm^3^ K mol^–1^ for **[1]^–^** is in agreement with the expected value of 14.17 cm^3^ K mol^–1^ for one isolated Dy^3+^ ion (^6^H_15/2_, *S* = 5/2, *L* = 5, *g* = 4/3). Upon cooling, the *χ*_M_*T* decreases gradually, then more rapidly below 130 K to reach a minimum of 12.55 cm^3^ K mol^–1^ at 2 K. This decrease is mainly attributed to the thermal depopulation of excited states. After one-electron oxidation of **[1]^–^** to form **1**, an increase in the room temperature *χ*_M_*T* value to 14.36 cm^3^ K mol^–1^ is observed. This increase is in accordance with the presence of a single Dy^3+^ ion and an additional non-interacting *S* = 1/2 site (low-spin Fe^3+^, 0.375 cm^3^ K mol^–1^ expected for *g* = 2 but actual g-value likely different). Upon decreasing the temperature, the *χ*_M_*T* value of **1** decreases more rapidly than **[1]^–^** below 60 K, reaching a minimum of 10.13 cm^3^ K mol^–1^ at 2 K. The more rapid decrease could be indicative of thermal depopulation of Stark sublevels or the presence of intra- and/or inter-molecular antiferromagnetic interactions.^47^

For the Er^3+^ analogues, the room temperature *χ*_M_*T* value for **[2]^–^** is 10.82 cm^3^ K mol^–1^. This value is only slightly lower than the expected value of 11.28 cm^3^ K mol^–1^ for a single isolated Er^3+^ ion (^4^I_15/2_, *S* = 3/2, *L* = 6, *g* = 6/5). After one-electron oxidation to **2**, the room temperature *χ*_M_*T* value increases to 11.43 cm^3^ K mol^–1^, corresponding to one Er^3+^ ion and one non-interacting Fe^3+^ ion. As observed in the analogous Dy^3+^ compounds, there is a more rapid decrease in the *χ*_M_*T* of the oxidized mixed-valent compound **2** at low temperatures.

The field dependence of the magnetization (*M*) was measured for each compound with fields up to 70 kOe (7 T) over a temperature range of 2–8 K (Fig. S3–S10†). In the Dy^3+^ compound **[1]^–^** at 2 K, the magnetization increases sharply until 1 T, followed by a more gradual increase, reaching a saturated moment of 5.34 *μ*_B_ at 7 T (Fig. S3†). This is near to the expected value of 5.23 *μ*_B_ for one uncorrelated Dy^3+^ ion in the presence of ligand field effects.^48^ The non-superposition in the temperature dependence (*M vs. H*/*T*) suggests the presence of significant magnetic anisotropy in **[1]^–^** (Fig. S4†). In **1** at 2 K, the magnetization increases sharply until 1 T and then gradually increases, reaching an unsaturated moment of 5.07 *μ*_B_ at 7 T (Fig. S5†). The lack of obvious saturation along with the non-superposition in the *M vs. H*/*T* data suggests the presence of low-lying excited states and/or significant anisotropy (Fig. S6†).^47^ The Er^3+^ compounds **[2]^–^** and **2** reach unsaturated moments at 7 T of 4.97 *μ*_B_ and 4.86 *μ*_B_, respectively (Fig. S7 and S9†). These unsaturated moments are in accordance with previously characterized mononuclear Er^3+^ complexes.^49^–^51^ The lack of clear saturation in the *M vs. H* data along with the non-superposition of *M vs. H*/*T* (Fig. S8 and S10†), implies low-lying excited states and/or magnetic anisotropy.

#### Dynamic magnetic properties and redox switchability

**Fig. 4:**
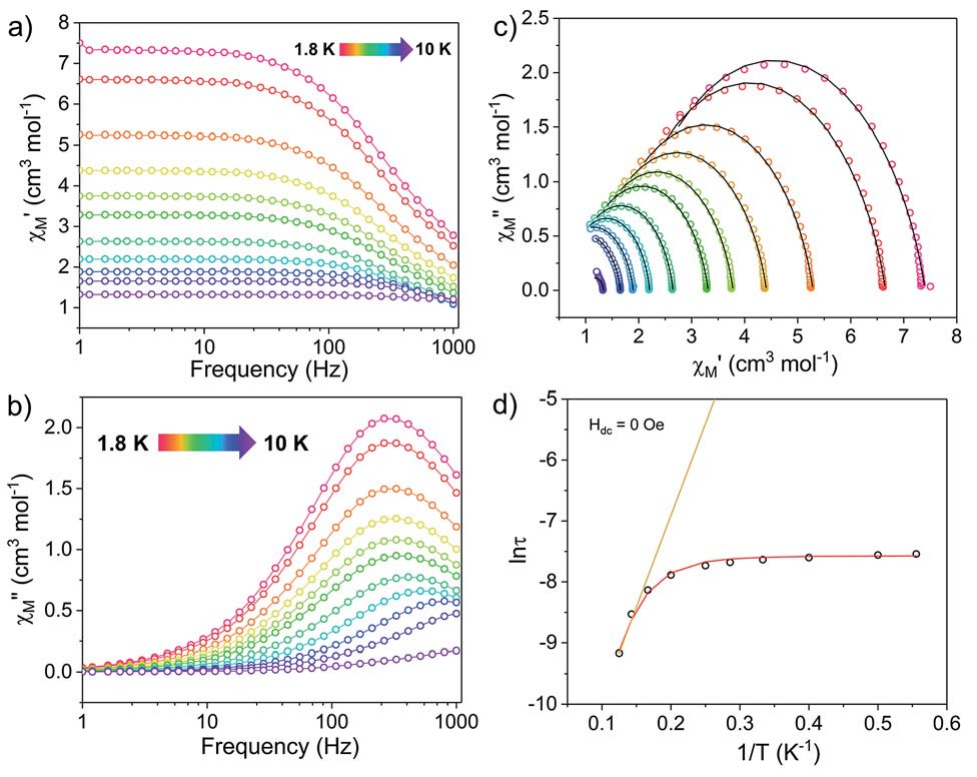
Ac susceptibility measurements for **[1]^–^** under zero dc field. (Left) Frequency dependence of the (a) in-phase, *χ*′, and (b) out-of-phase, *χ*′′, components of the ac susceptibility. (c) Cole–Cole plots, open circles are experimental data and black lines are fits to the generalized Debye equation.^52^ (d) Arrhenius plot, open circles are experimental data points. The orange line represents fit of the linear region, using the three highest temperature points, to the equation using the expression *τ*^–1^ = *τ*_0_^–1^ exp(–*U*_eff_/*k*_B_*T*); with *U*_eff_ = 20.9 cm^–1^ and *τ*_0_ = 2.43 × 10^–6^ s. The red curve represents the fit to eqn (1), with *U*_eff_ = 27.3(8) cm^–1^ and *τ*_0_ = 1.63(2) × 10^–6^ s.

Dynamic magnetic properties were investigated using alternating-current (ac) measurements. In **[1]^–^**, a signal in the out-of-phase component (*χ*′′) of the ac susceptibility was observed under zero applied dc field, indicative of slow magnetic relaxation (Fig. 4b). Cole–Cole (semi-circle) plots of the in-phase (*χ*′) *vs.* out-of-phase (*χ*′′) components of the ac susceptibility were fit to a generalized Debye model^10^,^52^ to extract relaxation times, *τ* (Fig. 4c). The natural logarithm of *τ* was plotted *vs.* the inverse temperature to construct the corresponding Arrhenius plot (Fig. 4d). The large temperature independent region observed at low temperatures in the *χ*′′ and Arrhenius plots of **[1]^–^** implies prevalent quantum tunnelling of the magnetization (QTM), a relaxation process that proceeds without the input of thermal energy.

The Arrhenius plot of **[1]^–^** (Fig. 4d) was fit using least squares regression to a model that accounted for multiple relaxation processes, including, QTM, Raman, and Orbach processes (eqn (1)).1
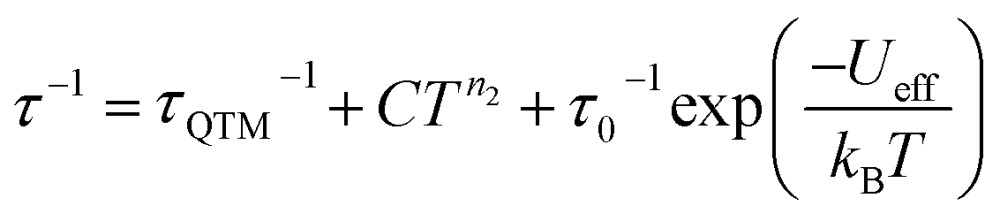



The parameters *τ*_QTM_, *C* (Raman coefficient), *τ*_0_ (pre-exponential factor), and *U*_eff_ (thermal barrier for Orbach relaxation) were treated as free-fit parameters. For Kramers ions, parameter *n*_2_ in the Raman pathway is expected to be 9; although allowing *n*_2_ to be a free-fit parameter resulted in a value of *n* = 5. A value lower than 9 may be expected in systems with low-lying excited states if optical phonons are taken into consideration.^53^,^54^ Only the Orbach process, the “over the barrier” pathway, appears linear on the plot of ln *τ vs. T*^–1^.

Due to the predominance of quantum tunnelling of magnetization (QTM) at low temperatures, few temperature dependent points were obtained. However, a barrier height, *U*_eff_ = 27.3(8) cm^–1^ (*τ*_0_ = 1.63(2) × 10^–6^ s), for the thermally activated Orbach process was calculated for **[1]^–^** under *H*_dc_ = 0 Oe using eqn (1). All parameters of the fit are listed in Table S5.†

**Fig. 5:**
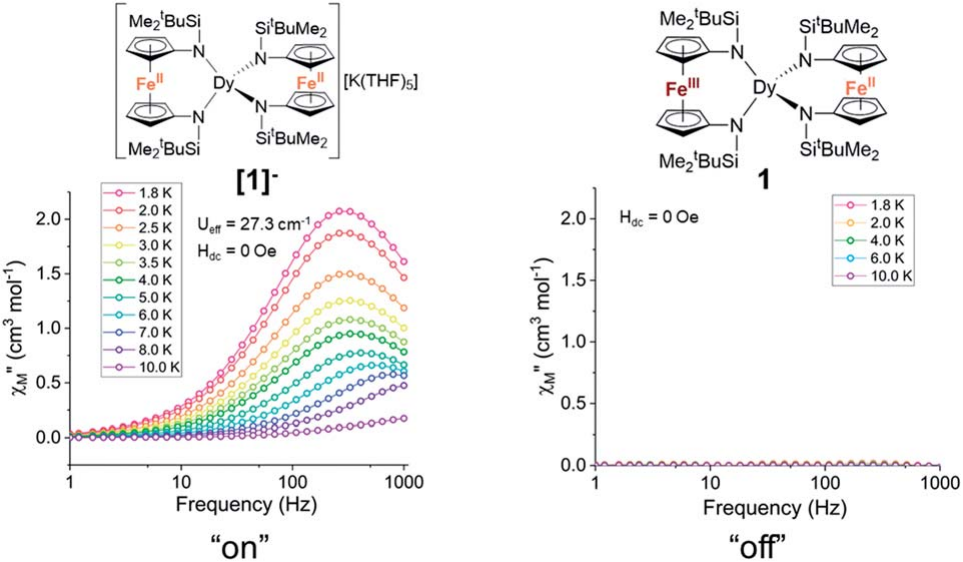
The one-electron oxidation of **[1]^–^** to **1** with half an equivalent of iodine results in redox switching of slow relaxation between “on” (**[1]^–^**, left) and “off” (**1**, right) modes under zero applied dc field. Lines are a guide for the eye.

Notably, the one-electron oxidation product **1** shows no evidence of slow relaxation under zero applied dc field at ac frequencies up to 1000 Hz (Fig. S11 and S12†). This is likely due to faster QTM in the oxidized species **1** than in the non-oxidized species **[1]^–^**. This could be attributed to both the lower symmetry around the Dy^3+^ ion in the mixed-valent compound and/or the introduction of an *S* = 1/2 site nearby the magnetically anisotropic Dy^3+^ ion. In detail, changes in ligand field and dipole-dipole interactions have been shown to facilitate mixing of magnetic states through which QTM can be introduced. Structural changes (and potentially packing effects) likely make the largest contribution to the change in magnetization dynamics. The appreciable loss of symmetry upon oxidation and change in ligand field would undoubtedly influence the orientation of the magnetic anisotropy axis and therefore the magnetization dynamics. In the absence of applied dc fields, the changes in magnetization dynamics upon reversible one-electron oxidation of **[1]^–^** to **1** can be thought of as “on”/“off” switching of the slow magnetic relaxation (frequencies up to 1000 Hz) (Fig. 5).

For both Er^3+^ compounds, **[2]^–^** and **2**, no signal was observed in the out-of-phase component (*χ*′′) of the ac susceptibility under zero dc field at ac frequencies up to 1000 Hz (Fig. S14 and S16†); likely a result of efficient ground state QTM processes for these species.

QTM between orthogonal Kramers ground states is typically caused by a perturbation Hamiltonian that allows for mixing of energetically close lying states. Examples of such perturbations include the presence of transverse anisotropy and dipole–dipole interactions between paramagnetic metal centres.^55^ To mitigate QTM in this series of compounds, various dc fields were applied during ac susceptibility measurements. Application of the static dc field lifts the degeneracy of the Kramers states, thereby reducing QTM. In the presence of a dc field, compounds **[1]^–^**, **1**, and **[2]^–^** show clear evidence of slow relaxation of the magnetization, with a signal in the out-of-phase component (*χ*′′) observed within the ac frequency range of 1 to 1000 Hz (Fig. S18, S22 and S26†). Notably, the oxidized mixed-valent Er^3+^ compound **2** displayed no evidence of slow relaxation at any investigated temperature or field (Fig. S30†).

**Fig. 6:**
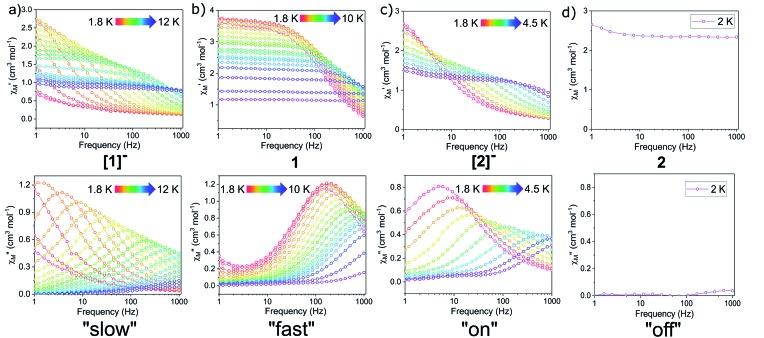
Frequency dependences of the in-phase, *χ*′, (top) and out-of-phase, *χ*′′, (bottom) components of the ac susceptibility for (a) **[1]^–^** with a 1000 Oe dc field (b) **1** with a 1000 Oe dc field (c) **[2]^–^** with a 500 Oe dc field (d) **2** with a 500 Oe dc field. Lines are guide for the eye.

**Fig. 7:**
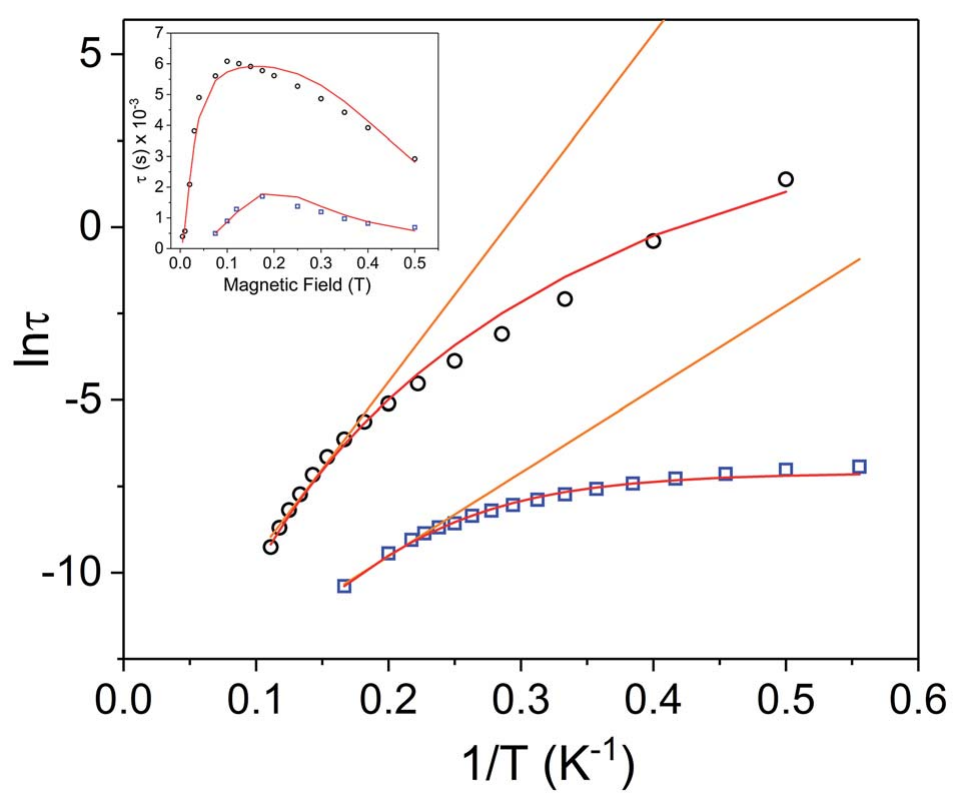
Arrhenius plots for Dy^3+^ complexes **[1]^–^** (black circles) and **1** (blue squares) under 1000 Oe applied dc field. The orange lines represent fits of the linear regions to the expression *τ*^–1^ = *τ*_0_^–1^ exp(–*U*_eff_/*k*_B_*T*) (Orbach only); resulting in *U*_eff_ values of 35.0 cm^–1^ (*τ*_0_ = 4.79 × 10^–7^ s) for **[1]^–^** and 16.8 cm^–1^ (*τ*_0_ = 5.79 × 10^–7^ s) for **1**. Red lines represent fits of the entire temperature region to eqn (2), giving *U*_eff_ values of 46(2) cm^–1^ (*τ*_0_ = 7.3(7) × 10^–7^ s) for **[1]^–^** and 27.2(5) cm^–1^ (5.0(4) × 10^–7^ s) for **1**. Inset: field dependence of the relaxation times (*τ*) for **[1]^–^** (black circles) and **1** (blue squares), red lines are fits to eqn (3). See Table S5† for all fitting parameters.

In the variable field ac measurements for **[1]^–^** at 5 K, a transition from a faster relaxation process to a slower relaxation process is observed around 300 Oe (Fig. S18†). An optimal static field of 1000 Oe was determined from the maximum (slowest relaxation) in the plot of the field dependence of *τ* (Fig. 7, inset, Fig. S20†). Therefore, variable temperature ac susceptibility measurements for **[1]^–^** were collected with a dc field of 1000 Oe (Fig. 6a). At 1000 Oe, the maxima of the out-of-phase signals (*χ*′′) are shifted to lower frequencies relative to the zero field measurements, as the reduction of QTM results in slower magnetic relaxation. Furthermore, with the application of the 1000 Oe dc field, the Arrhenius plot displays temperature dependence over the entire temperature regime (Fig. 7).

Arrhenius plots (ln *τ vs. T*^–1^) for the applied field measurements were fit using least squares regression to a model that accounted for multiple relaxation pathways, including direct, QTM, Raman, and Orbach (eqn (2)).2
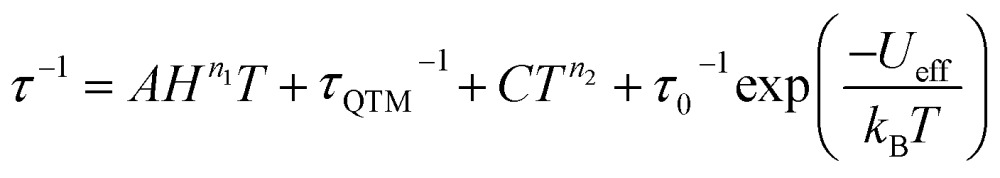



To avoid over-parameterization during fitting, the field dependence of *τ* was initially fit for the two field dependent processes, direct and QTM, to obtain the direct relaxation parameter *A* and the QTM parameters *B*_1_ and *B*_2_, according to eqn (3).^53^ Typically, *n*_1_ = 4 for Kramers ions in the absence of hyperfine interactions.^54^ Parameter *D* was added to account for the field independent contributions from Raman and Orbach relaxation processes.^53^ Parameters *A*, *B*_1_, *B*_2_, and *D* were treated as free fit parameters. All fitting parameters are listed in Tables S5 and S6.† For **[1]^–^** at 5 K, the very gradual decrease in *τ* values at fields above 1000 Oe suggests minimal contributions of single phonon direction relaxation mechanisms (Fig. 7, inset, Fig. S20†).^53^ At fields below 1000 Oe, the increase in *τ* with increasing field was modelled successfully by the QTM term.3
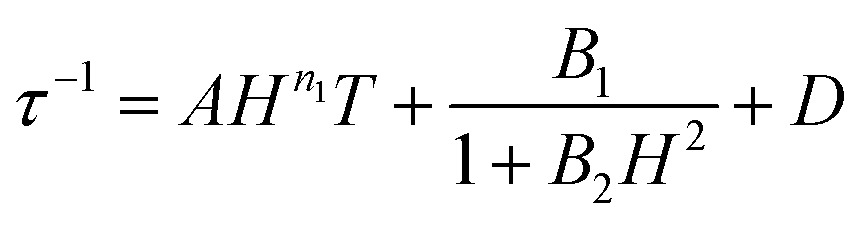



The parameters obtained from eqn (3) were held fixed while fitting the temperature dependent Arrhenius plots (ln *τ vs. T*^–1^), according to eqn (2). For **[1]^–^**, the temperature dependence of *τ* was fit to direct, Raman, and Orbach relaxation processes (Fig. 7). Allowing the Raman exponent *n*_2_ to be a free fit parameter resulted in a value of *n*_2_ = 7. For Kramers ions, an *n*_2_ value of 9 is expected; however, lower *n*_2_ values may be anticipated if optical phonons are taken into consideration.^53^ A barrier height of *U*_eff_ = 46(2) cm^–1^ (*τ*_0_ = 7.3(7) × 10^–7^ s) was obtained; 18.7 cm^–1^ larger than the barrier calculated under zero dc field.

Variable field ac measurements for **1** at 2 K are displayed in Fig. S21–S22.† The field dependence of *τ* was fit for both direct and QTM processes according to eqn (3) (Fig. 7 (inset), Fig. S24†). A value of *n*_1_ = 2 was used to obtain a fit, corresponding to a Kramers ion in a hyperfine field.^54^,^56^ The maximum *τ* value (slowest relaxation) occurs at a field of 1750 Oe at 2 K. However, to maintain consistency with the ac measurements for **[1]^–^**, variable temperature ac measurements for **1** were carried out using a 1000 Oe dc field (Fig. 6b). The maxima of *χ*′′ for the mixed-valent compound **1** are shifted to higher frequencies relative to **[1]^–^**, implying faster magnetic relaxation for a given temperature of the mixed valent compound **1** (Fig. 6b). The Arrhenius plot was fit using eqn (2), accounting for direct, Raman, QTM and, Orbach processes, to give *U*_eff_ = 27.2(5) cm^–1^ (*τ*_0_ = 5.0(4) × 10^–7^ s) (Fig. 7). Notably, the *U*_eff_ for the mixed-valent species **1** is 18.8 cm^–1^ lower than the non-oxidized species **[1]^–^**. All fitting parameters for **1** are listed in Table S5.†

Redox switchability of the dynamic magnetic properties is best illustrated by comparing the relaxation times in the Arrhenius plot of **[1]^–^** and **1** (Fig. 7): the one-electron oxidation of **[1]^–^** to **1** results in faster relaxation times at a given temperature and a lower *U*_eff_ value.

**Fig. 8:**
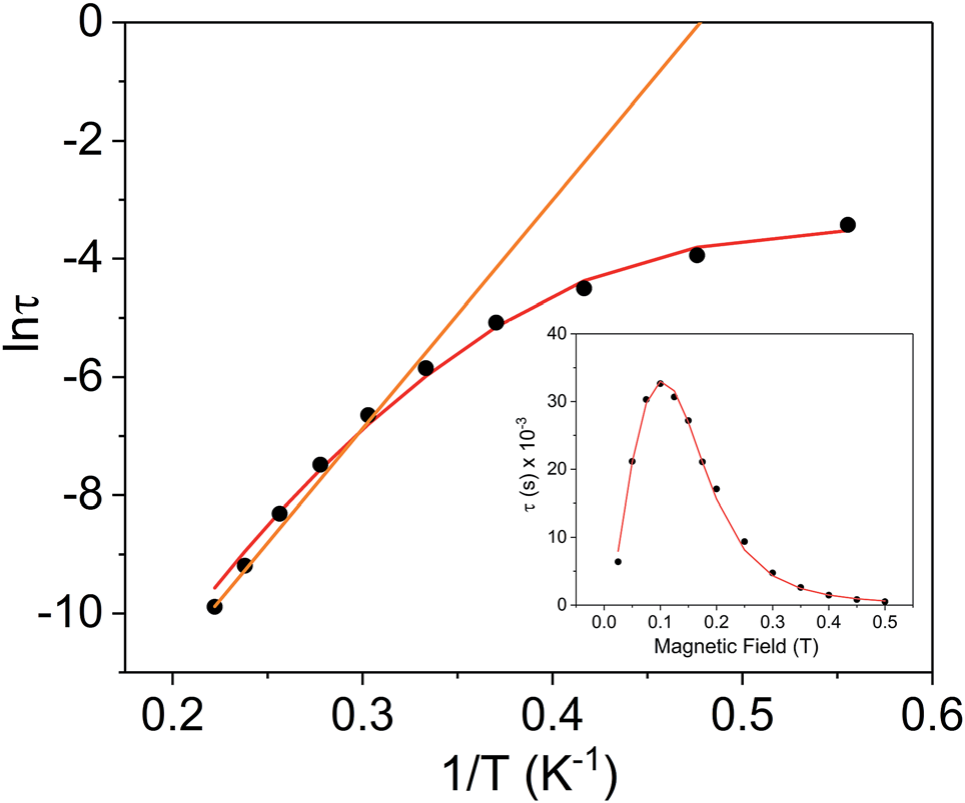
Arrhenius plot for the Er^3+^ complex **[2]^–^** (black circles) under a 500 Oe applied dc field. The orange line represents the fit of the linear region (six highest temperature points) to the expression *τ*^–1^ = *τ*_0_^–1^ exp(–*U*_eff_/*k*_B_*T*) (Orbach only); resulting in a *U*_eff_ value of 26.9 cm^–1^ (*τ*_0_ = 9.52 × 10^–9^ s). The red line represents fit of the entire temperature region to eqn (2), giving a *U*_eff_ value of 29(2) cm^–1^ (*τ*_0_ = 4(1) × 10^–7^ s). Inset: field dependence of the relaxation times (*τ*) for **[2]^–^** (black circles), red line is fit to eqn (3). See Table S6† for all fitting parameters.

Using the above described methodology, variable-field ac measurements were collected for the Er^3+^ compound **[2]^–^** at 2 K (Fig. S25–S27†). The field dependence of *τ* was successfully fit using eqn (3) (Fig. 8 (inset), Fig. S28, Table S6†). Variable temperature ac susceptibility data were collected using an applied field of 500 Oe (Fig. 6c). The corresponding relaxation times were fit over the entire temperature region of the Arrhenius plot (Fig. 8), according to eqn (2), resulting in an extracted value of *U*_eff_ = 29(2) cm^–1^ (*τ*_0_ = 4(1) × 10^–7^ s). The one-electron oxidized species, **2**, did not display any signs of slow magnetic relaxation under dc fields as high as 1500 Oe in ac experiments (Fig. S30†).

In summarizing the magnetic results, we find that the prolate Er^3+^ ion in the **[2]^–^**/**2** system allows solely for reversible “on”/“off” switching of the slow magnetic relaxation (in the presence of a small dc field) (Fig. 6c and d), while the oblate Dy^3+^ ion in the **[1]^–^**/**1** redox system enables bi-functional redox switchability of magnetic properties: “on”/“off” (no dc field) (Fig. 5) and “slow”/“fast” (with dc field) (Fig. 6a, b and 7).

The distinct shapes of f-electron density of Dy^3+^ (oblate) and Er^3+^ (prolate) result in very different magnetic anisotropy axes and therefore different magnetization dynamics under the same ligand field and molecular symmetry conditions. Previously, isostructural Dy^3+^ and Er^3+^ complexes have been shown to exhibit different magnetization dynamics. In the four-coordinate, trigonal pyramidal series of compounds [Li(thf)_4_][Ln{N(SiMe_3_)_2_}_3_Cl]·2 thf, the Er^3+^ analogue displayed SMM behaviour under zero dc field, whereas the Dy^3+^ analogue exhibited only field induced SMM behaviour.^57^–^59^ This difference was mainly attributed to the local symmetry and orientation of the magnetic anisotropic axis. The trigonal pyramidal geometry was found to not be ideal for either oblate or prolate ions but was relatively more favourable for prolate type ions, such as Er^3+^.^59^

The coordination geometry of the bis-diamidoferrocene complexes presented here was found to be relatively more favourable for the oblate Dy^3+^ ion than for the prolate Er^3+^ ion. The differences in behaviour between the Dy^3+^ and Er^3+^ complexes are attributed to the orientation of the anisotropy axis under the same ligand field conditions due to the difference in f-electron density. We believe the largest contributor to the change in magnetization dynamics upon oxidation to be the change in ligand field and lowering of local symmetry. In detail, the oxidation of one of the diamidoferrocene ligands (in going from **[1]^–^** and **[2]^–^** to **1** and **2**) results in inequivalent binding of the two ligands to the central lanthanide ion. Additionally, packing effects and the presence or absence of counter ions may contribute to the structural changes.

The magnetic anisotropy axis of **1** was determined utilizing a quantitative electrostatic model for the prediction of the orientation of the ground state anisotropy axis in Dy^3+^ compounds described by Chilton *et al.* (MAGELLAN).^60^ Considering charged ligands as point charges, the method minimizes electrostatic repulsion between the point charges and f-electron density. Using the molecular structure of **1**, the anisotropy axis was determined under three different scenarios: (1) assigning both Fe centres as neutral, (2) assigning a +1 charge to the Fe centre closer to the Dy^3+^ ion, and (3) assigning a +1 charge to the Fe centre further from the Dy^3+^ ion (Fig. S35†). The addition and location of the +1 point charge did not lead to considerable differences in the orientation of the anisotropic axis (Fig. S35†). The location of the negative point charges (amide ligands) was found to be the largest contributor, with the magnetic anisotropy axis aligned in the same plane as the shorter Dy–N bonds.

#### Cyclic voltammetry for **[1]^–^**/**1** and **[2]^–^**/**2**

**Fig. 9:**
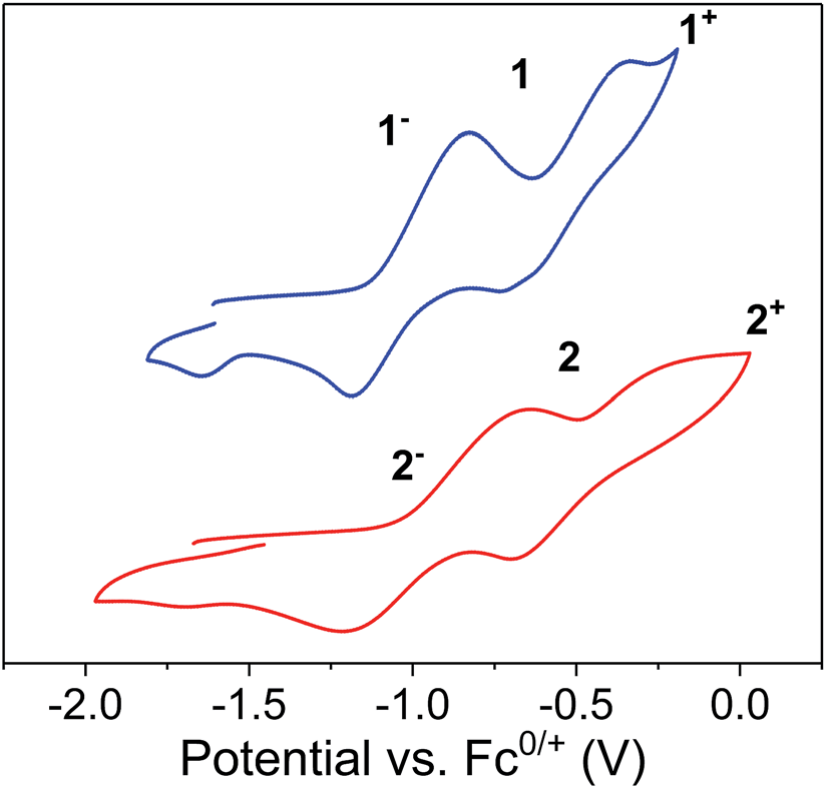
Cyclic voltammograms of **[1]^–^** (top) and **[2]^–^** (bottom) at 200 mV s^–1^ in thf with 0.1 M Bu_4_NPF_6_ as electrolyte. Referenced *versus* Cp_2_Fe^0/+^.

The separation in electrochemical potentials between individual redox couples of multi-redox systems can provide a useful initial estimate for the presence of electronic communication in mixed-valent species. The cyclic voltammogram of a solution of **[1]^–^** in thf, displays two quasi-reversible redox processes centred at *E*_1/2_ =–1.00 and –0.540 V *vs.* [Cp_2_Fe]^0/1+^ (Fig. 9, top/blue). These processes correspond to the Fe^2/3+^ redox couples of the two individual ferrocene diamide ligands. The cyclic voltammogram of the free ligand exhibits one reversible redox process at –0.60 V *vs.* [Cp_2_Fe]^0/1+^.^39^ The potential separation of the two processes in **[1]^–^** is Δ*E*_1/2_ = 0.460 V, corresponding to a comproportionation constant of *K*_c_ = 5.96 × 10^7^. The cyclic voltammogram of a solution of **[2]^–^** in thf displays two reversible redox processes centred at *E*_1/2_ = –0.930 and –0.410 V *vs.* [Cp_2_Fe]^0/1+^ (Fig. 9, bottom/red), corresponding to *K*_c_ = 6.16 × 10^8^. The large *K*_c_ values in **[1]^–^** and **[2]^–^** suggest considerable electronic interaction between the two iron sites in solution and is consistent with a Robin and Day Class II classification.^61^ These values are significantly large but smaller than the values observed for the U^4+^ analogue reported by Diaconescu *et al.* (Δ*E*_1/2_ = 1.25 V, *K*_c_ = 1.55 × 10^21^).^39^ Importantly, these observations are consistent with the formulation that the central *f*-block element is indeed critically involved in the electronic communication between the mixed-valent iron ions, resulting in larger electronic coupling for the actinide ion (U^4+^) than for the trivalent lanthanide ions (Dy^3+^, Er^3+^), featuring the more contracted frontier orbitals.

#### ^57^Fe Mössbauer spectroscopy and UV-vis-NIR spectroscopy

**Fig. 10:**
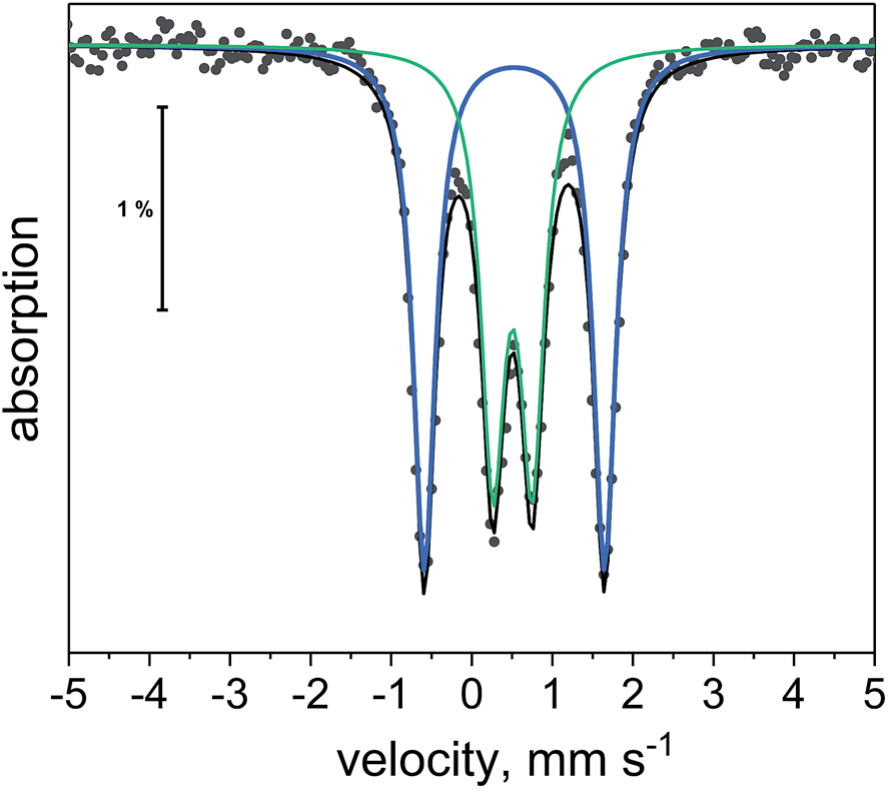
^57^Fe Mössbauer spectrum of **1** at 5 K. Black dots are experimental points. Black line is overall two-site fit. Blue and green lines are the individual sub-spectra for the two-site fit.

We utilized ^57^Fe Mössbauer spectroscopy to establish the presence of electronic communication of the mixed-valent iron ions in **1** in the solid state. In the ^57^Fe Mössbauer spectrum of the all ferrous species **[1]^–^** in zero field, a single characteristic doublet was observed, corresponding to the spectroscopically identical low-spin Fe^2+^ ions in **[1]^–^**, with an isomer shift (*δ*) of 0.54 mm s^–1^ and quadrupole splitting (Δ*E*_Q_) of 2.34 mm s^–1^ (Fig. S36†). The ^57^Fe Mössbauer spectrum of the mixed-valent compound **1** exhibited two doublets (Fig. 10). The spectrum could in principle be fit using both a two-site (Fig. 10) model (two doublets) or a three-site model (Fig. S37†) (one doublet, two singlets). Given the chemical nature of **1**, we believe that only the two-site model is appropriate here. As such, we fit the data using one doublet corresponding to the Fe^2+^ ion (*δ* = 0.528 mm s^–1^; Δ*E*_Q_ = 2.238 mm s^–1^) and an equally contributing second doublet corresponding to the Fe^3+^ ion (*δ* = 0.506 mm s^–1^; Δ*E*_Q_ = 0.490 mm s^–1^). The Mössbauer spectrum is consistent with a trapped valent system over the temperature range studied (5 K to 150 K; Fig. S38†), in which electron transfer is slower than the time scale of Mössbauer spectroscopy (∼10^–7^ s^–1^). However, electronic communication between Fe^2+^ and Fe^3+^ is clearly present, as the signal corresponding to the ferric site is quadrupole split. In the absence of electronic communication this signal would be expected to occur as a singlet.^41^ Furthermore, the smaller Δ*E*_Q_ observed for the ferrous site in **1** as compared to **[1]^–^** is also consistent with the formulation of Fe^2+^···Fe^3+^ electronic communication. Similar spectroscopic signatures in mixed-valent dinuclear iron complexes have previously been reported for [Cp*_2_Fe_2_(as-indacene)]˙^+^.^62^

We utilized UV-vis-NIR absorption spectroscopy to probe the degree of electronic communication in solution. A broad band associated with an intervalence charge transfer (IVCT) transition was observed in the near-IR region of dilute thf solutions of the mixed-valent compounds **1** and **2** at *λ*_max_ = 1056 nm and 1043 nm, respectively (Fig. S40 and S42†). This broad, low-energy IVCT band is indicative of electronic communication in solution and is consistent with a Class II system,^63^ supporting the observations from CV and ^57^Fe Mössbauer spectroscopy. Moreover, in solution, intermolecular communication is assumed to be negligible, indicating the presence of intramolecular electronic communication in these mixed valent systems. The UV-vis-NIR absorption spectra of **[1]^–^** and **[2]^–^** in thf displayed no bands in the NIR region (Fig. S39 and S41†).

## Conclusions

Our studies highlight the utility of diamidoferrocene ligands in the construction of redox switchable SMMs. The high chemical reversibility of the Fe^2+/3+^ redox couples can be exploited to alter magnetization dynamics of Dy^3+^ and Er^3+^ based SMMs. The mixed valent Fe ions in complexes **1** and **2** feature electronic communication with each other. Importantly, redox-switchable “on”/“off” and “slow”/“fast” SMM behaviour can be obtained depending on the experimental conditions and the nature of the four-coordinate lanthanide ion. The combined results of this comprehensive molecular level study are important contributions towards the development of rational molecular design guidelines for future switchable magnetic molecules and materials.

## Experimental section

### General considerations

All reactions and manipulations were carried out under anaerobic and anhydrous conditions in an argon filled glovebox (Vigor). All syntheses and manipulations were carried out using disposable plastic spatulas. Tetrahydrofuran (thf), hexanes, toluene and diethyl ether were dried and deoxygenated using a solvent purification system (JC Meyer Solvent Systems) and were stored over molecular sieves in an argon-filled glovebox. Anhydrous dysprosium(iii) iodide and iodine were purchased from Alfa Aesar. Eicosane was purchased from Acros Organics. Sublimed anhydrous erbium(iii) iodide was generously donated by Prof. Tim Hughbanks' group (Texas A&M). fc[HNSi(*t*-Bu)Me_2_]_2_ was prepared as previously described.^39^ Benzyl potassium was prepared *via* the deprotonation of toluene by nBuLi/KO^*t*^Bu as described previously.^64^ UV-vis-NIR spectra were recorded using a Shimadzu SolidSpec-3700 spectrophotometer over a range of 300 nm to 2000 nm and matched screw-capped quartz cuvettes. Elemental analyses were carried out by Midwest Microlab (Indianapolis, IN).

### Experimental procedures

#### Preparation of [K_2_(OEt_2_)]fc[NSi(*t*-Bu)Me_2_]_2_

Performed *via* a modification of a previous procedure.^39^ fc[HNSi(*t*-Bu)Me_2_]_2_ (314.9 mg, 0.7083 mmol) was dissolved in diethyl ether (8 ml) and cooled in a freezer (–30 °C) for 30 min. Solid benzyl potassium (186.2 mg, 1.430 mmol) was added to the cold stirring solution of fc[HNSi(*t*-Bu)Me_2_]_2_ in diethyl ether. The reaction mixture was allowed to warm up to rt and stirred for 2 h. All solvent was removed *in vacuo*. The remaining orange solid was washed with hexanes and collected on a medium frit. Solid was dried *in vacuo* and used without further purification (369.4 mg, 88%).

#### Preparation of K(thf)_5_[Dy(fc[NSi(*t*-Bu)Me_2_]_2_)_2_] **[1]^–^**

[K_2_(OEt_2_)]fc[NSi(*t*-Bu)Me_2_]_2_ (127 mg, 0.213 mmol) was dissolved in 3 ml of tetrahydrofuran and cooled in the freezer (–30 °C) for 30 min. To a separate vial, dysprosium(iii) iodide (58.0 mg, 0.107 mmol) and thf (3 ml) were added and the solution was cooled in the freezer (–30 °C) for 30 min. The [K_2_(OEt_2_)]fc[NSi(*t*-Bu)Me_2_]_2_ solution was added dropwise to the stirring DyI_3_ suspension while cold. The reaction mixture was stirred for 1.5 h at rt. All volatiles were removed *in vacuo* and the solid was washed with hexanes and dried. A minimal amount of thf was added and the solution was filtered through Celite. Removal of the thf *in vacuo* yielded a yellow-orange solid. Air sensitive yellow-orange plate crystals were obtained from a concentrated thf solution layered with hexanes at –30 °C for 24 h (117 mg, 76%). Unit cell parameters (110 K): *a* = 16.500(2) Å, *b* = 19.510(3) Å, *c* = 12.286(2) Å, *α* = 90°, *β* = 90°, *γ* = 90°, V = 3955 Å^3^. Anal. calcd for C_64_H_116_N_4_DyFe_2_KSi_4_O_5_ (**[1]^–^**): C, 53.11; H, 8.08; N, 3.87. Found: C, 52.62; H, 7.77; N, 3.59.

#### Preparation of K(thf)_5_[Er(fc[NSi(*t*-Bu)Me_2_]_2_)_2_] **[2]^–^**

Same procedure as in the synthesis of **[1]^–^**, with [K_2_(OEt_2_)]fc[NSi(*t*-Bu)Me_2_]_2_ (135 mg, 0.227 mmol), erbium(iii) iodide (62.2 mg, 0.113 mmol) and 8 ml of tetrahydrofuran. Single crystals were obtained from a concentrated thf solution layered with hexanes in the freezer (–30 °C) for 24 h (117 mg, 71%) of very air-sensitive yellow-orange plate crystals. Unit cell parameters (110 K): *a* = 16.443(3) Å, *b* = 18.088(4) Å, *c* = 12.828(3) Å, *α* = 90°, *β* = 90°, *γ* = 90°, V = 3815 Å^3^. Anal. Calcd for C_56_H_100_N_4_ErFe_2_KSi_4_O_3_ (**[2]^–^** – 2 thf): C, 51.4; H, 7.71; N, 4.28. Found: C, 51.32; H, 7.50; N, 3.86.

#### Preparation of Dy(fc[NSi(*t*-Bu)Me_2_]_2_)_2_ (**1**)

K(thf)_5_[Dy(fc[NSi(*t*-Bu)Me_2_]_2_)_2_] (75.6 mg, 0.0522 mmol) and diethyl ether (5 ml) were added to a vial and placed in the freezer (–30 °C) for 30 min. To a separate vial, iodine (6.8 mg, 0.027 mmol) and diethyl ether (5 ml) were added and placed in the freezer (–30 °C) for 30 min. The iodine solution was added to the stirring K(thf)_5_[Dy(fc[NSi(*t*-Bu)Me_2_]_2_)_2_] solution while cold, resulting in immediate colour change to dark red. The reaction mixture was stirred for 40 min at rt. All volatiles were removed *in vacuo*. The resulting dark solid was extracted into hexanes and filtered through Celite. The volatiles were removed *in vacuo*, yielding a dark purple solid. X-ray quality dark red-purple block crystals were grown from a concentrated hexanes solution after 24 h at –30 °C (27.7 mg, 51%). Unit cell parameters (110 K): *Pbca*, *a* = 20.025(4) Å, *b* = 19.663(4) Å, *c* = 24.499(5) Å, *α* = 90°, *β* = 90°, *γ* = 90°, V = 9646 Å^3^. Anal. calcd for C_44_H_76_N_4_DyFe_2_Si_4_ (1): C, 50.44; H, 7.31; N, 5.35%. Found: C, 50.17; H, 6.88; N, 5.30%.

#### Preparation of Er(fc[NSi(*t*-Bu)Me_2_]_2_)_2_ (**2**)

The same procedure as for **1** was followed using K(thf)_5_[Er(fc[NSi(*t*-Bu)Me_2_]_2_)_2_] (101.8 mg, 0.07011 mmol), iodine (8.5 mg, 0.034 mmol) and diethyl ether (10 ml). X-ray quality air sensitive dark red-orange block crystals were grown from a concentrated hexanes solution after 24 h at –30 °C (39.6 mg, 55%). Unit cell parameters (110 K): *Pbca*, *a* = 19.794(2) Å, *b* = 19.822(2) Å, *c* = 24.603(2) Å, *α* = 90°, *β* = 90°, *γ* = 90°, V = 9653 Å^3^: Anal. calcd. for C_44_H_76_N_4_ErFe_2_Si_4_ (2): C, 50.22; H, 7.28; N, 5.32%. Found: C, 49.99; H, 7.24; N, 5.20%.

### X-ray structure determination

Crystals suitable for X-ray diffraction were mounted on a nylon loop and placed in a cold N_2_ stream (Oxford) maintained at 110 K. A BRUKER APEX 2 Duo X-ray (three-circle) diffractometer was used for crystal screening, unit cell determination, and data collection. The X-ray radiation employed was generated from a Mo sealed X-ray tube (*K*_α_ = 0.70173 Å with a potential of 40 kV and a current of 40 mA). Bruker AXS APEX II software was used for data collection and reduction. Absorption corrections were applied using the program SADABS.^65^ A solution was obtained using XT/XS in APEX2.^66^–^69^ Hydrogen atoms were placed in idealized positions and were set riding on the respective parent atoms. All non-hydrogen atoms were refined with anisotropic thermal parameters. Absence of additional symmetry and voids were confirmed using PLATON (ADDSYM).^70^,^71^ The structure was refined (weighted least squares refinement on *F*^2^) to convergence.^68^,^72^ For **1**, the Si4(C39–C44) group was found disordered between two positions and was modeled successfully with an occupancy ratio of 0.55 : 0.45.

### Magnetic measurements

A representative procedure for the preparation of the samples for magnetic characterization is as follows. Crystalline sample was crushed up into a fine powder before loading into a high purity 7 mm NMR tube (Norell). A layer of eicosane was added to the tube, covering the sample. The tube was then flame sealed under vacuum. To restrain the sample, the sealed tube was placed in a water bath (39 °C) until the eicosane melted and was evenly distributed throughout the sample. The sample was loaded into a straw affixed to the end of the sample rod. Magnetic measurements were carried out using a Quantum Design MPMS 3 SQUID magnetometer (TAMU Vice President of Research). Dc susceptibility measurements were carried out over a temperature range of 1.8 to 300 K. Ac measurements were carried out using a 2 Oe switching field. Data was corrected for diamagnetic contributions from the straw, sample tube, eicosane and core diamagnetism using Pascal's constants.^73^ Cole–Cole plots were fitted to the generalized Debye equation using least-squares regression.^52^ Arrhenius plots and tau *vs. H* plots were fit using least squares regression.

### ^57^Fe Mössbauer spectroscopy

Crystalline samples were loaded into a Teflon cup inside a glovebox and covered with a layer of paraffin oil. The cup was brought out of the glovebox and immediately stored frozen in liquid nitrogen until measured. Mössbauer spectra were collected on a model MS4 WRC low-field, variable temperature spectrometer (See Co., Edina, MN). Zero magnetic field spectra were obtained by removing the 500 G magnets from the exterior of the instrument. Temperatures were varied using a temperature controller on the heating coil on the sample holder. The instrument was calibrated using an α-Fe foil at room temperature. Obtained spectra were fitted using WMOSS software (See Co.).

### Electrochemistry

Cyclic voltammograms were measured in an argon filled glovebox (Vigor). Data were collected using a Gamry Instruments Reference 600 potentiostat with Gamry Framework software. Glassy carbon working electrode, 1 mm diameter Pt wire counter electrode, and silver-wire pseudo-reference electrode were used. Scan rates of 100 mV s^–1^ to 250 mV s^–1^ were used. Ferrocene was added at the end of each data collection and the ferrocene/ferrocenium couple was used as an internal standard.

## Conflicts of interest

There are no conflicts to declare.

## Supplementary Material

Supplementary information

Crystal structure data
