# Supplementary material for: Transition metal redox switches for reversible “on/off” and “slow/fast” single-molecule magnet behaviour in dysprosium and erbium bis-diamidoferrocene complexes
Source: Chem Sci. 2017 Oct 2;8(12):8039–49. doi: 10.1039/c7sc03380j (PMC5853775; doi:10.1039/c7sc03380j)
Supplement: Supplementary file 1 [file SC-008-C7SC03380J-s001.pdf]

Electronic Supplementary Material (ESI) for Chemical Science.
This journal is © The Royal Society of Chemistry 2017

Electronic Supplementary Information for

Transition Metal Redox Switches for Reversible "On/Off" and "Slow/Fast" Single-Molecule Magnet Behaviour in Dysprosium and Erbium bis-Diamidoferrocene Complexes

Courtney M. Dickie[a] Alexander L. Laughlin[b], Joshua D. Wofford[a], Nattamai S. Bhuvanesh[a] and Michael Nippe[a] *

[a] Department of Chemistry, Texas A&M University, 3255 TAMU, College Station, TX, 77843 (USA)

[b] Department of Chemistry and Biochemistry, University of California, Los Angeles, 607 Charles E. Young Drive East, Los Angeles, California 90095, United States.

Table of Contents

**Table S1**	Crystallographic data for **1**	S5
**Table S2**	Crystallographic data for **2**	S6
**Table S3**	Selected geometric parameters for compound **1**	S7
**Tables S4**	Selected geometric parameters for compound **2**	S7
**Figure S1**	Molecular structures of **1** and **2** with Ln···Fe distances	S8
**Figure S2**	Unit cell packing in **1** highlighting intermolecular Fe···Fe contacts	S9
**Figure S3**	Field dependence of the magnetization for [**1**]$^-$.	S10
**Figure S4**	Field dependence, temperature dependence of the magnetization for [**1**]$^-$.	S10
**Figure S5**	Field dependence of the magnetization for **1**.	S11
**Figure S6**	Field dependence, temperature dependence of the magnetization for **1**.	S11
**Figure S7**	Field dependence of the magnetization for [**2**]$^-$.	S12
**Figure S8**	Field dependence, temperature dependence of the magnetization for [**2**]$^-$.	S12
**Figure S9**	Field dependence of the magnetization for **2**.	S13
**Figure S10**	Field dependence, temperature dependence of the magnetization for **2**.	S13
**Table S5**	Ac susceptibility data fitting parameters for the Dy^{3+} compounds **[1]**$^-$ and **1**	S14
**Table S6**	Ac susceptibility data fitting parameters for the Er^{3+} compound **[2]**$^-$	S14
**Figure S11**	Frequency dependence of the in-phase component (χ') of the ac susceptibility for **1** under zero dc field.	S15
**Figure S12**	Frequency dependence of the out-of-phase component (χ") of the ac susceptibility for **1** under zero dc field.	S15
**Figure S13**	Frequency dependence of the in-phase (χ') component of the ac susceptibility for **[2]**$^-$ under zero dc field.	S16

**Figure S14**	Frequency dependence of the out-of-phase component (χ”) of the ac susceptibility for **[2]**⁻ under zero dc field.	S16
**Figure S15**	Frequency dependence of the in-phase (χ’) component of the ac susceptibility for **2** under zero dc field.	S17
**Figure S16**	Frequency dependence of the out-of-phase component (χ”) of the ac susceptibility for **2** under zero dc field.	S17
**Figure S17**	Frequency dependence of the in-phase component (χ’) of the ac susceptibility for **[1]**⁻ at T = 5 K with applied dc fields varying from 50 to 5000 Oe.	S18
**Figure S18**	**.** Frequency dependence of the out-of-phase component (χ”) of the ac susceptibility for **[1]**⁻ at T = 5 K with various dc fields.	S18
**Figure S19**	Cole-Cole plots for **[1]**⁻ at 5 K with various applied dc fields.	S19
**Figure S20**	Field dependence of the relaxation times (τ) in **[1]**⁻	S19
**Figure S21**	Frequency dependence of the in-phase component (χ’) of the ac susceptibility for **1** at T = 2 K with various dc fields.	S20
**Figure S22**	Frequency dependence of the out-of-phase component (χ”) of the ac susceptibility for **1** at T = 2 K with various dc fields.	S20
**Figure S23**	Cole-Cole plots for **1** at 2 K with various applied dc fields.	S21
**Figure S24**	Field dependence of the relaxation times (τ) in **1**.	S21
**Figure S25**	Frequency dependence of the in-phase component (χ’) of the ac susceptibility for **[2]**⁻ at 2 K with various dc fields.	S22
**Figure S26**	Frequency dependence of the out-of-phase component (χ”) of the ac susceptibility for **[2]**⁻ at 2 K with various dc fields.	S22
**Figure S27**	Cole-Cole plots for **[2]**⁻ at 2 K with various applied dc fields.	S23
**Figure S28**	Field dependence of the relaxation times (τ) in **[2]**⁻.	S23
**Figure S29**	Frequency dependence of the in-phase component (χ’) of the ac susceptibility for **2** at 2 K with various dc fields.	S24
**Figure S30**	Frequency dependence of the out-of-phase component (χ”) of the ac susceptibility for **2** at 2 K with various dc fields.	S24
**Figure S31**	Temperature dependence of the out-of-phase component (χ”) of the ac susceptibility for **[1]**⁻ with a 1000 Oe applied dc field.	S25
**Figure S32**	Cole-Cole plots for **[1]**⁻, with an applied dc field of 1000 Oe	S25

**Figure S33**	Cole-Cole plots for **1**, with an applied dc field of 1000 Oe.	S26
**Figure S34**	Cole-Cole plots for **[2]**$^-$, with an applied dc field of 500 Oe.	S26
**Figure S35**	Predicted orientations of the magnetic anisotropy axes in **1** under three scenarios using MAGELLAN	S27
**Figure S36**	^{57}Fe Mössbauer spectrum of **[1]**$^-$ at10 K with no external field.	S28
**Figure S37**	^{57}Fe Mössbauer spectrum of **1**, at 5 K with overall and sub-spectra for the three-site fit.	S28
**Figure S38**	^{57}Fe Mössbauer spectrum of **1** at 5 K, 50 K and 150 K.	S29
**Figure S39**	UV-vis-NIR spectrum of **[1]**$^-$ in thf.	S30
**Figure S40**	UV-vis-NIR spectrum of **1** in thf.	S30
**Figure S41**	UV-vis-NIR spectrum of **[2]**$^-$ in thf.	S31
**Figure S42**	UV-vis-NIR spectrum of **1** in thf.	S32

Table S1. Crystallographic data for **1**

Compound	**Dy(fc[NSi(t-Bu)Me$_2$]$_2$)$_2$ (1)**
Formula	$DyFe_2N_4Si_4C_{44}H_{76}$
Formula weight	
Crystal size (mm)	0.34 x 0.22 x 0.114
T (K)	110
Λ	0.71073
Crystal system	Orthorhombic
Space group	Pbca
a, Å	20.025(4)
b, Å	19.663(4)
c, Å	24.499(5)
α, °	90
β, °	90
γ, °	90
Volume, Å^3	9646(3)
Z	8
ρ_{calcd}, Mg/m^3	1.443
F(000)	4336
Absorption coefficient (mm^{-1})	2.260
Θ_{min}, Θ_{max}, °	1.662, 24.998
Index ranges	-23 <= h <=23
	-23 <= k <=23
	-29 <= l <= 29
Reflections collected	89008
Independent reflections	8495 [R(int) = 0.0859]
Completeness to Θ = 24.998 °	100 %
Absorption correction	Semi-empirical from equivalents
Refinement method	Full-matrix least-squares on F^2
Data/restraints/parameters	8495 / 280 / 584
Goodness of fit on F^2	1.125
Final R indices [I > 2σ(I)]	R_1^a = 0.0376, wR_2^b = 0.0753
R indices (all data)	R_1^a = 0.0650, wR_2^b = 0.0947
Largest diff peak and hole, eÅ^3	1.093 and 0.902

$^a R_1 = 3||F_o|-|F_c||/3|F_o|$. $^b wR_2 = [3[w(F_o^2 - F_c^2)^2]/3[w(F_o^2)^2]]^{½}$, $w = 1/\sigma^2(F_o^2) + (aP)^2 + bP$, where $P = [\max(0$ or $F_o^2) + 2(F_c^2)]/3$.

Table 2. Crystallographic data for **2**

Compound	**Er(fc[NSi(t-Bu)Me$_2$]$_2$)$_2$ (1)**
Formula	$ErFe_2N_4Si_4C_{44}H_{76}$
Formula weight	
Crystal size (mm)	0.15 x 0.08 x 0.07
T (K)	110
λ	0.71073
Crystal system	Orthorhombic
Space group	Pbca
a, Å	19.794(2)
b, Å	19.822(2)
c, Å	24.603(2)
α, °	90
β, °	90
γ, °	90
Volume, Å^3	9653(2)
Z	8
ρ_{calcd}, Mg/m^3	1.448
F(000)	4352
Absorption coefficient (mm^{-1})	2.449
Θ_{min}, Θ_{max}, °	1.673, 19.999
Index ranges	-19 <= h <=19
	-19 <= k <=19
	-23 <= l <= 23
Reflections collected	81679
Independent reflections	4502
Absorption correction	Semi-empirical from equivalents
Refinement method	Full-matrix least-squares on F^2
Data/restraints/parameters	4502 / 1008 / 516
Goodness of fit on F^2	
Final R indices [I > 2σ(I)]	$R_1^a = 0.0742$, $wR_2^b = 0.1491$
R indices (all data)	$R_1^a = 0.1587$, $wR_2^b = 0.1847$
Largest diff peak and hole, eÅ^3	1.353 and 1.273

$^aR_1 = 3||F_o|-|F_c||/3|F_o|$. $^bwR_2 = [3[w(F_o^2 - F_c^2)^2]/3[w(F_o^2)^2]]^{½}$, $w = 1/\sigma^2(F_o^2) + (aP)^2 + bP$, where $P = [\max(0$ or $F_o^2) + 2(F_c^2)]/3$.

Table S3. Selected geometric parameters for compound **1**.

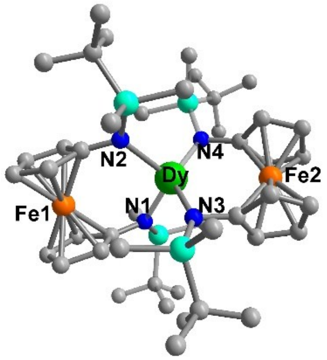

distances		
	Dy···Fe1, Å	3.792(2)
	Dy···Fe2, Å	3.368(2)
	Dy-N1, Å	2.370(4)
	Dy-N2, Å	2.338(4)
	Dy-N3, Å	2.262(4)
	Dy-N4, Å	2.252(4)
angles		
	N1-Dy-N2, °	110.5(2)
	N1-Dy-N3, °	104.2(2)
	N1-Dy-N4, °	102.7(2)
	N2-Dy-N3, °	100.3(2)
	N2-Dy-N4, °	106.5(2)
	N3-Dy-N4, °	131.9(2)

Table S4. Selected geometric parameters for compound **2**.

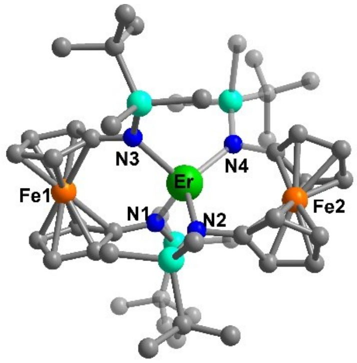

distances		
	Er···Fe1, Å	3.819(5)
	Er···Fe2, Å	3.498(4)
	Er-N1, Å	2.27(1)
	Er-N2, Å	2.22(2)
	Er-N3, Å	2.29(1)
	Er-N4, Å	2.21(2)
angles		
	N1-Er-N2, °	104.8(6)
	N1-Er-N3, °	109.6(5)
	N1-Er-N4, °	104.2(6)
	N2-Er-N3, °	102.2(6)
	N2-Er-N4, °	126.7(6)
	N3-Er-N4, °	108.7(5)

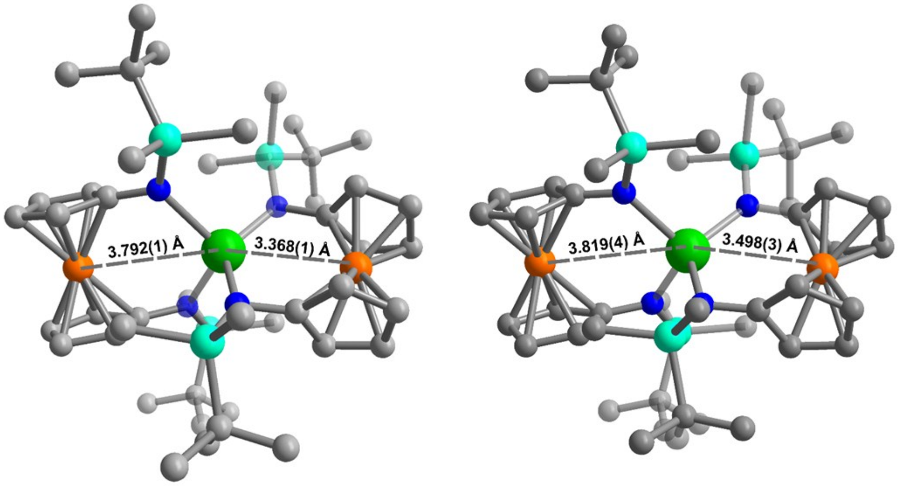

1 2

Figure S1. Molecular structure of Dy(fc[NSi(*t*-Bu)Me_2]$_2$)$_2$ **1** (left) and Er(fc[NSi(*t*-Bu)Me_2]$_2$)$_2$ **2** (right). Green = Ln, orange = Fe, cyan = Si, blue = N, grey = C. Hydrogen atoms omitted for clarity. Ln···Fe distances are highlighted: Dy···Fe = 3.792(1) Å and 3.368(1) Å, Er···Fe = 3.819(4) Å and 3.498(3) Å.

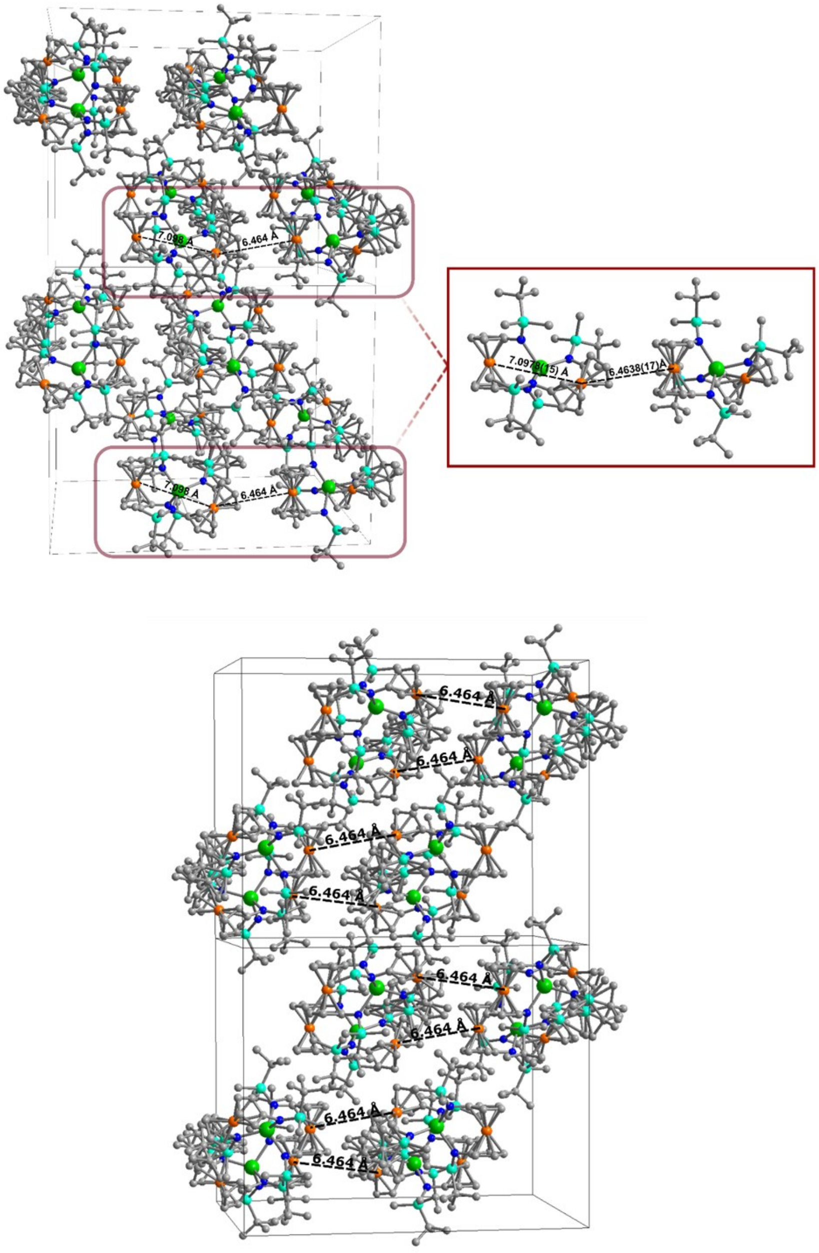

Figure S2. Unit cell packing in **1**. Top: the closest Fe···Fe contacts of 6.464(2) Å (intermolecular) and the longer Fe···Fe contacts of 7.098(2) Å (intramolecular) are highlighted. Bottom: the intermolecular Fe···Fe contacts are highlighted.

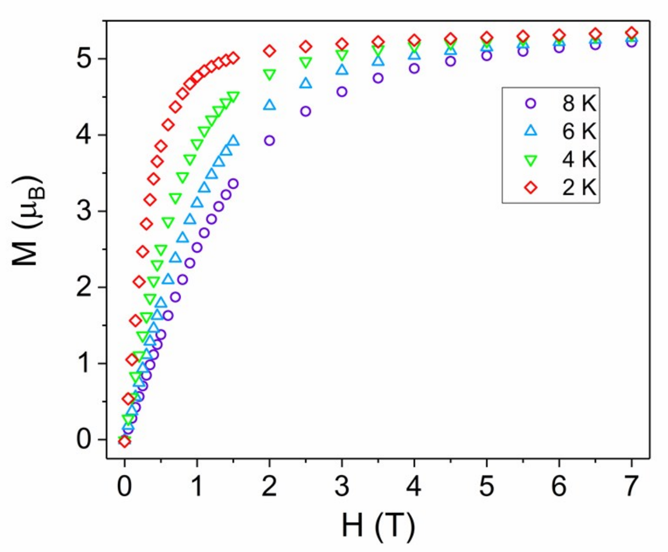

Figure S3. Field dependence of the magnetization for [**1**]$^-$.

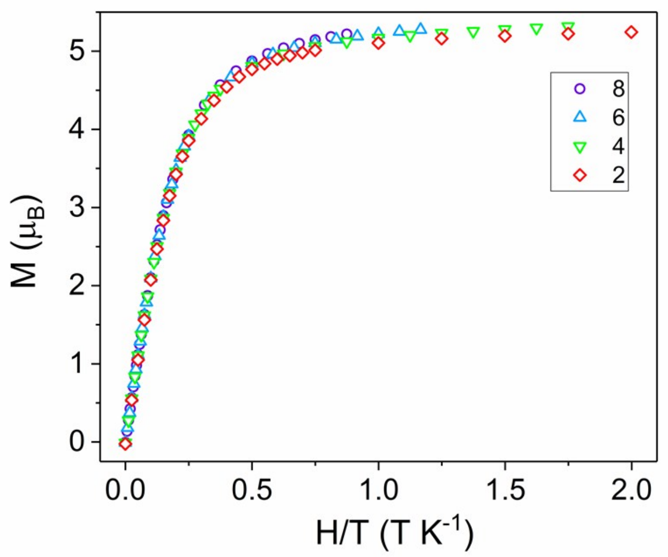

Figure S4. Field dependence, temperature dependence of the magnetization for **[1]**$^-$.

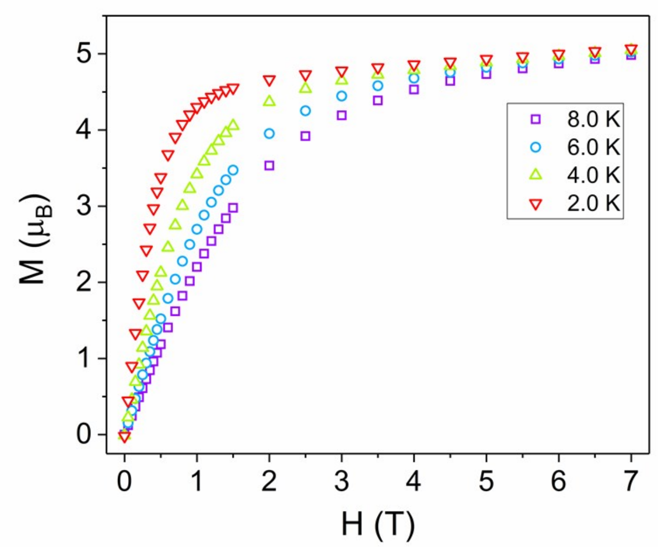

Figure S5. Field dependence of the magnetization for **1**.

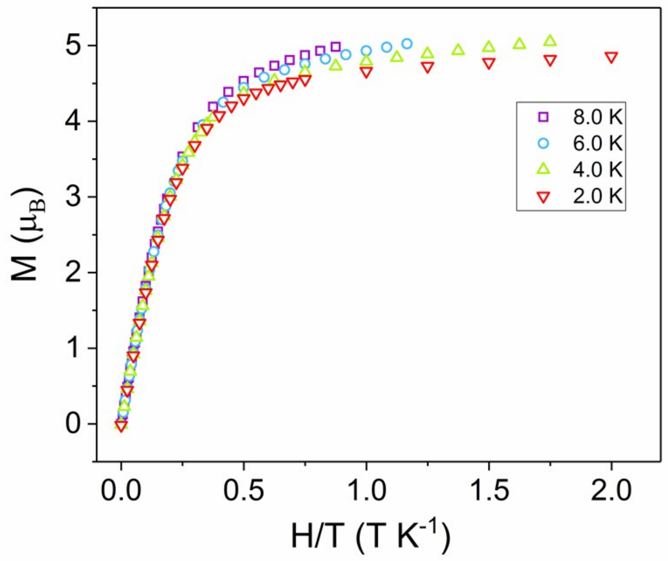

Figure S6. Field dependence, temperature dependence of the magnetization for **1**.

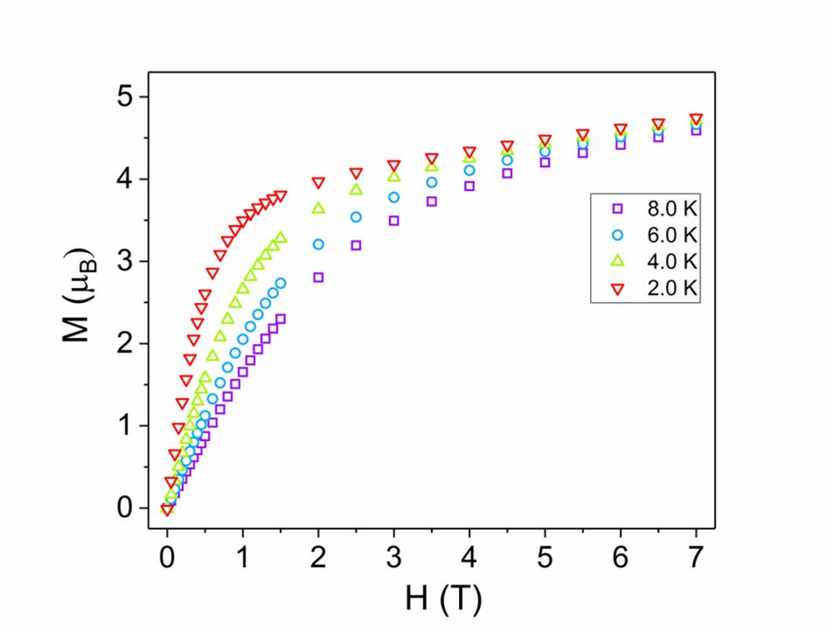

Figure S7. Field dependence of the magnetization for **[2]**$^-$.

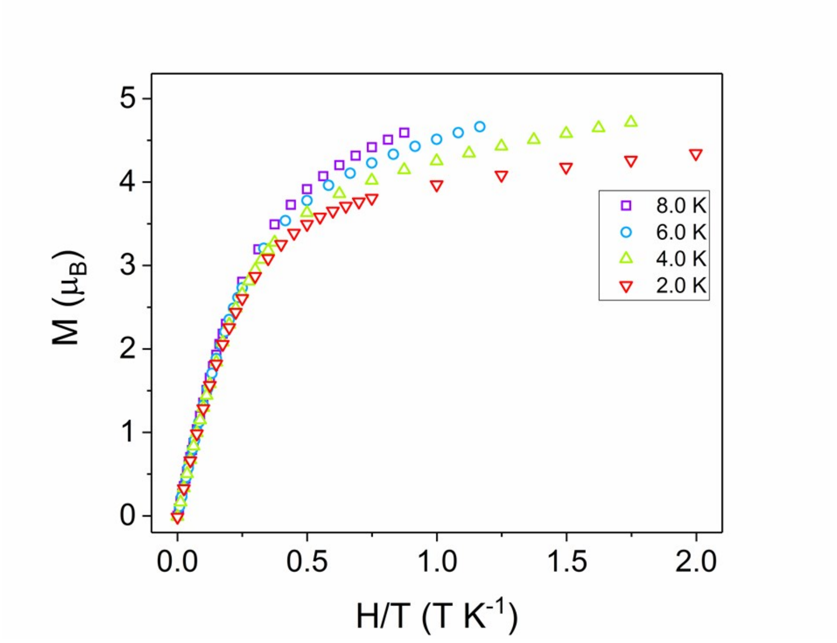

Figure S8. Field dependence, temperature dependence of the magnetization for **[2]**$^-$.

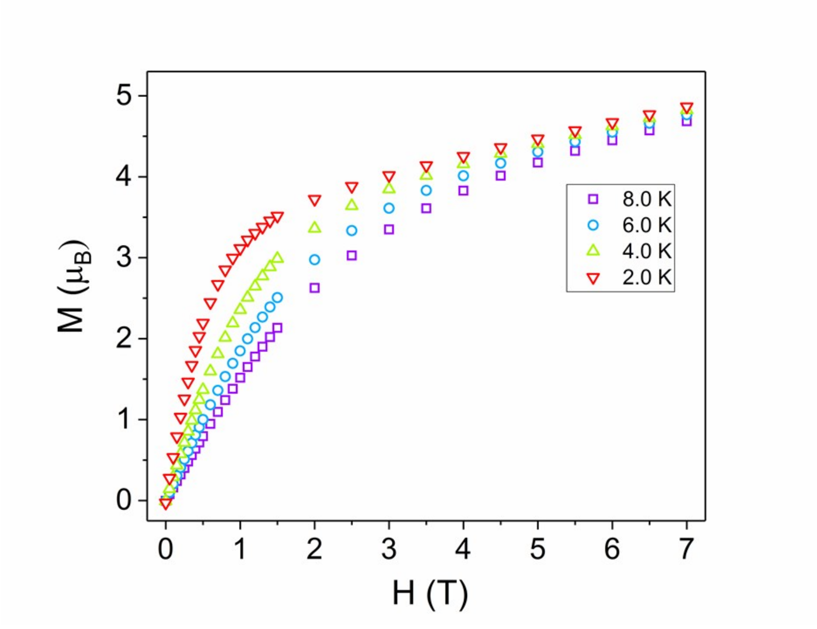

Figure S9. Field dependence of the magnetization for **2**.

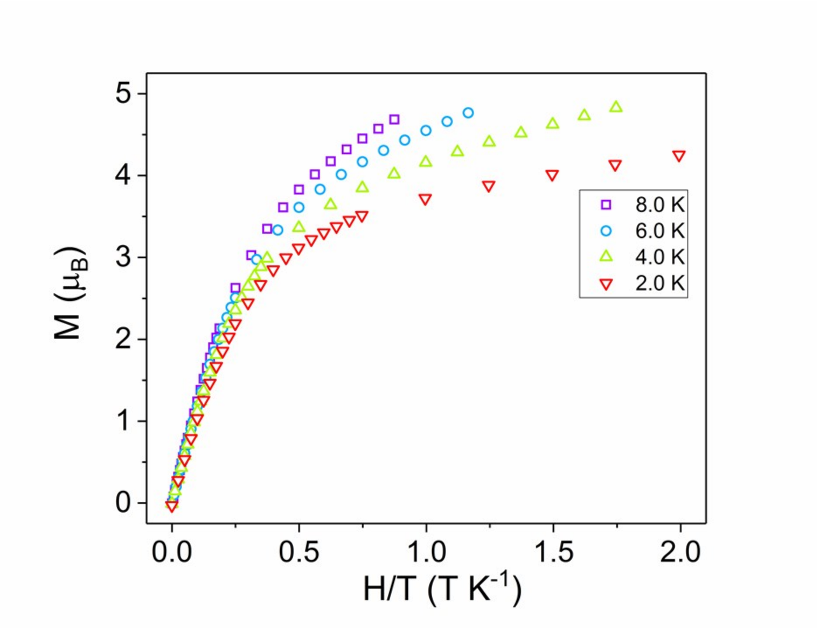

Figure S10. Field dependence, temperature dependence of the magnetization for **2**.

$$\tau^{-1} = \tau_0^{-1} exp\left(\frac{-U_{eff}}{kT}\right)$$ **(linear approximation)**

$$\tau^{-1} = \tau_{QTM}^{-1} + CT^{n_2} + \tau_0^{-1} exp\left(\frac{-U_{eff}}{kT}\right)$$ **(eqn 1)**

$$\tau^{-1} = AH^{n_1}T + \tau_{QTM}^{-1} + CT^{n_2} + \tau_0^{-1} exp\left(\frac{-U_{eff}}{kT}\right)$$ **(eqn 2)**

$$\tau^{-1} = AH^{n_1}T + \frac{B_1}{1 + B_2H^2} + D$$ **(eqn 3)**

Table S5. Fitting parameters for the Dy^{3+} compounds **[1]**$^-$ and **1**.

	K(thf)$_5$[Dy(fc[NSi(*t*-Bu)Me$_2$]$_2$)$_2$]<br>**[1]**$^-$				Dy(fc[NSi(*t*-Bu)Me$_2$]$_2$)$_2$<br>**1**	
**dc field (Oe)**	0		1000		1000	
**Approximation**	linear	eqn 1	linear	eqn 2/eqn 3	Linear	eqn 2/eqn 3
**A (s^{-1} T^{--n1} K^{-1})**	-	-	-	615	-	3.77 x 10^3
**n_1**	-	-	-	4	-	2
**B_1 (s^{-1})**	-	-	-	3.22 x 10^3	-	2.55 x 10^{14}
**B_2 (T^{-2})**	-	-	-	9.92 x 10^3	-	2.36 x 10^{13}
**D (s^{-1})**	-	-	-	162	-	0
**C (s^{-1} K^{-n2})**	-	0.08(1)	-	0.0018(3)	-	3.63(1)
**n_2**	-	5	-	7	-	5
**τ_{QTM} (s)**	-	5.03 x 10^{-4}	-	-	-	8.77 x 10^{-4}
**τ_0 (s)**	2.43 x 10^{-6}	1.63(2) x 10^{-6}	4.79 x 10^{-7}	7.3(7) x 10^{-7}	5.79 x 10^{-7}	5.0(4) x 10^{-7}
**U_{eff} (cm^{-1})**	**20.9**	**27.3(8)**	**35.0**	**46(2)**	**16.8**	**27.2(5)**

a) QTM terms were not included in the fitting of temperature dependence of [1]$^-$.

Table S6. Fitting parameters for the Er^{3+} compound **[2]**$^-$.

	K(thf)$_5$[Er(fc[NSi(*t*-Bu)Me$_2$]$_2$)$_2$]<br>**[2]**$^-$	
**dc field (Oe)**	500 Oe	
**Approximation**	Linear	eqn 2/eqn 3
**A (s^{-1} T^{--n1} K^{-1})**	-	1.29 x 10^4
**n_1**	-	4
**B_1 (s^{-1})**	-	4.63 x 10^4
**B_2 (T^{-2})**	-	70.8 x 10^5
**D (s^{-1})**	-	21.2
**C (s^{-1} K^{-n2})**	-	1.85(2) x 10^{-2}
**n_2**	-	9
**τ^{-1} $_{QTM}$**	-	3.33 x 10^{-2}
**τ_0 (s)**	9.52 x 10^{-9}	4(1) x 10^{-7}
**U_{eff} (cm^{-1})**	**26.9**	**29(2)**

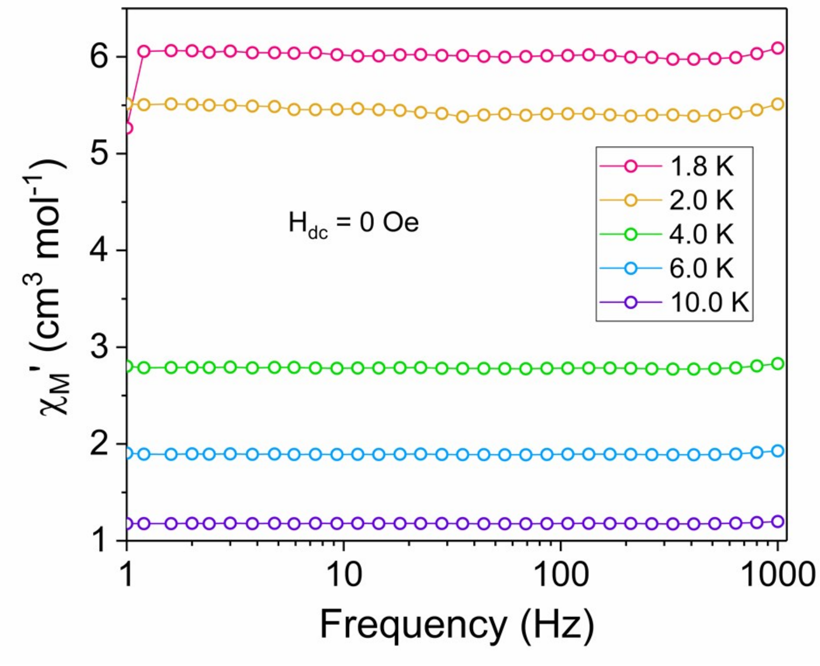

Figure S11. Frequency dependence of the in-phase component (χ’) of the ac susceptibility for **1** under zero dc field. Lines are a guide for the eye.

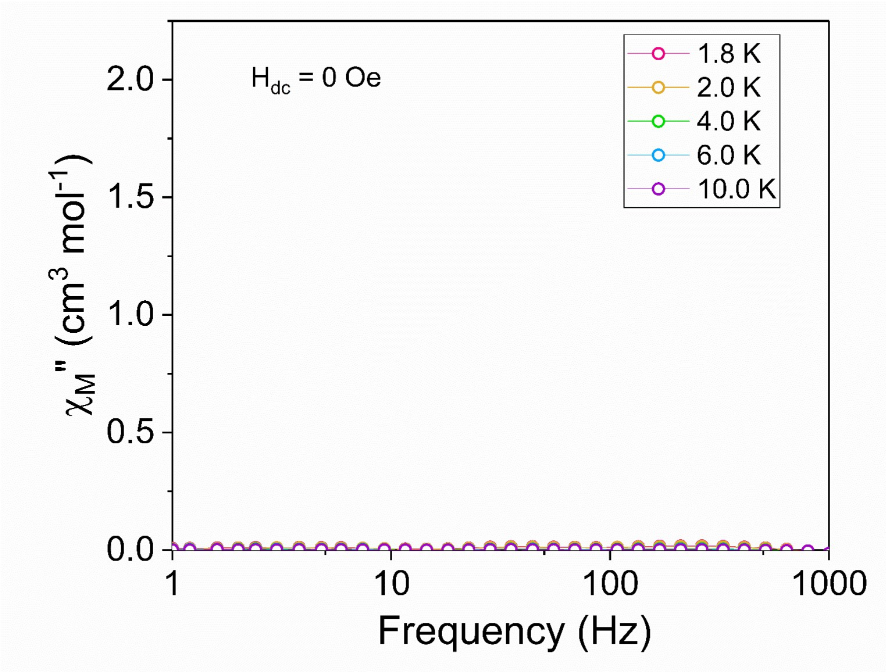

Figure S12. Frequency dependence of the out-of-phase component (χ”) of the ac susceptibility for **1** under zero dc field. Lines are a guide for the eye.

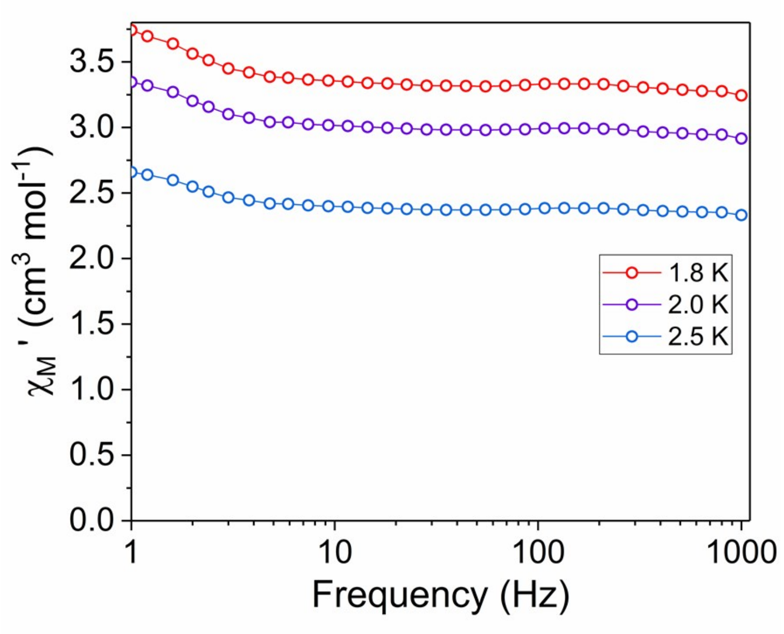

Figure S13. Frequency dependence of the in-phase (χ') component of the ac susceptibility for **[2]**⁻ under zero dc field (H_{dc} = 0 Oe). Lines are a guide for the eye.

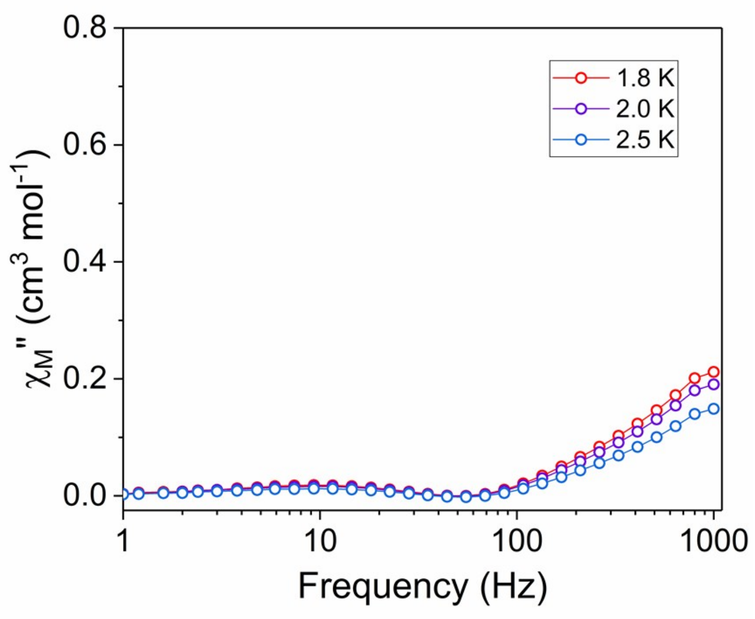

Figure S14. Frequency dependence of the out-of-phase component (χ") of the ac susceptibility for **[2]**⁻ under zero dc field (H_{dc} = 0 Oe). Lines are a guide for the eye.

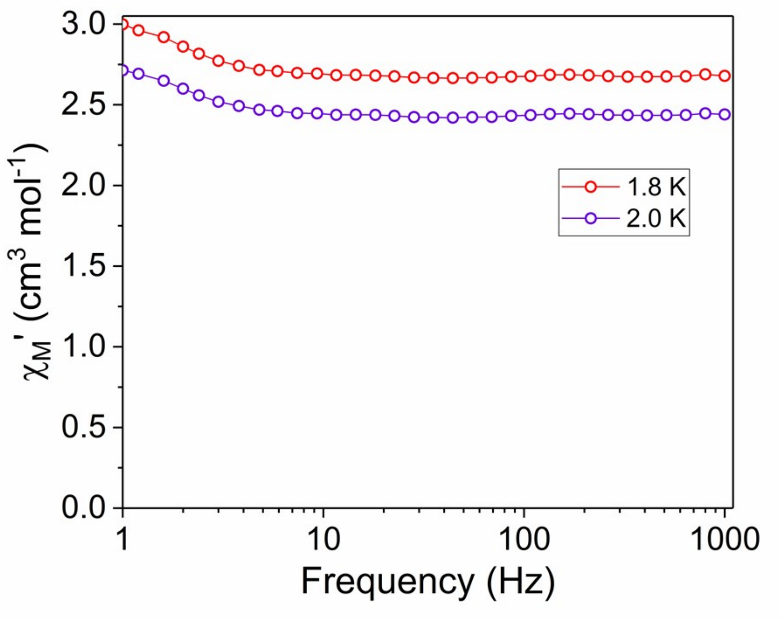

Figure S15. Frequency dependence of the in-phase (χ') component of the ac susceptibility for **2** under zero dc field (H_{dc} = 0 Oe). Lines are a guide for the eye.

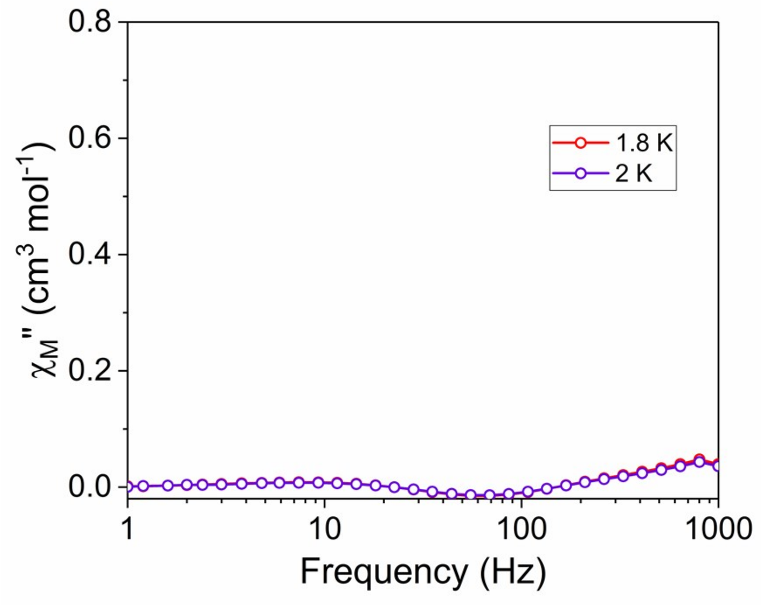

Figure S16. Frequency dependence of the out-of-phase component (χ") of the ac susceptibility for **2** under zero dc field (H_{dc} = 0 Oe). Lines are a guide for the eye.

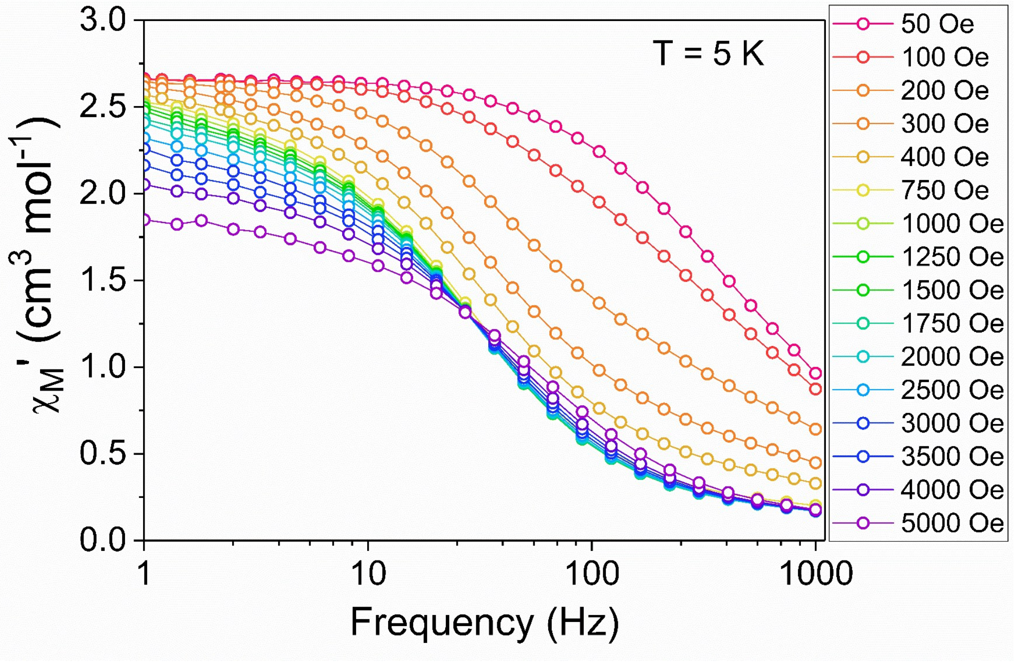

Figure S17. Frequency dependence of the in-phase component (χ') of the ac susceptibility for **[1]**$^-$ at T = 5 K with a 2 Oe switching field and applied dc fields varying from 50 to 5000 Oe. Lines are a guide for the eye.

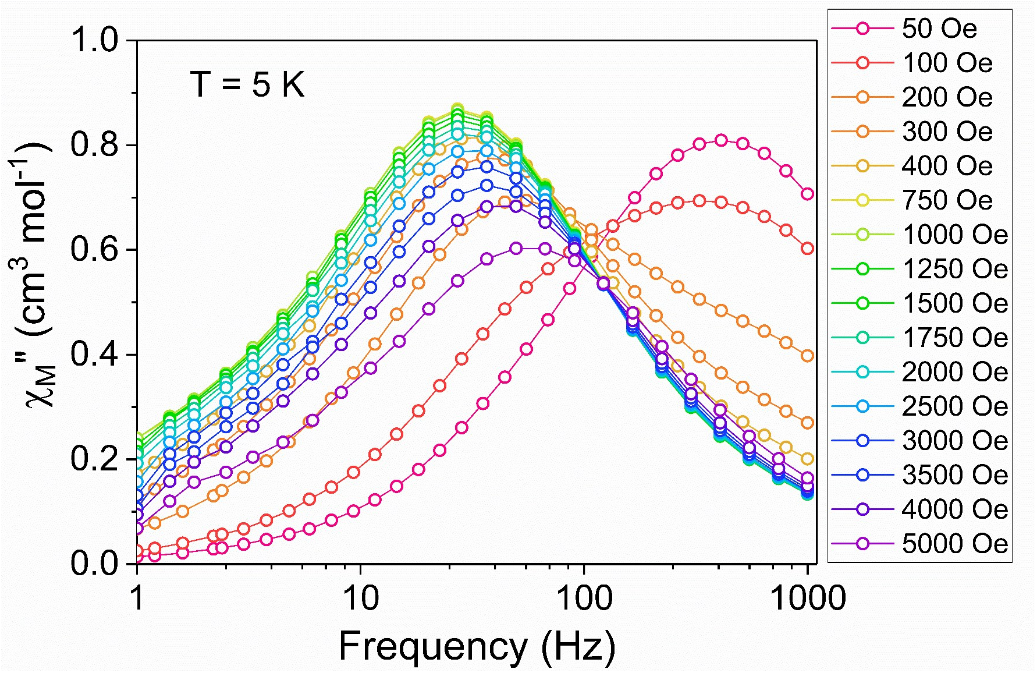

Figure S18. Frequency dependence of the out-of-phase component (χ") of the ac susceptibility for **[1]**$^-$ at T = 5 K with 2 Oe switching field and applied dc fields varying from 50 to 5000 Oe. Lines are a guide for the eye.

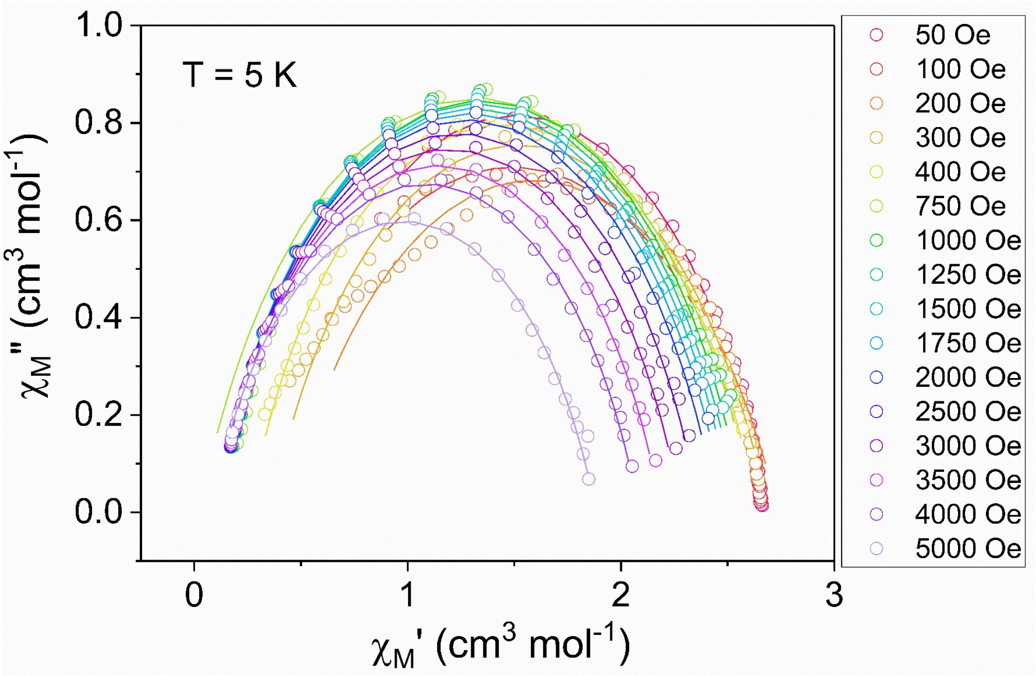

Figure S19. Cole-Cole plots for **[1]**$^-$ at 5 K with various applied dc fields. Open circles are experimental data, lines are fits to the generalized Debye equation.

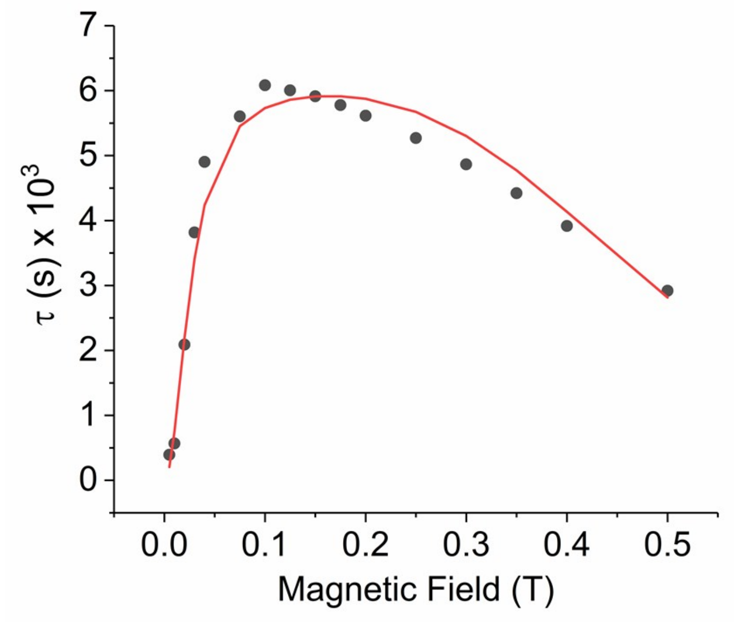

Figure S20. Field dependence of the relaxation times (τ) in **[1]**$^-$. Black circles are experimental data points, red line represents the fit to eqn 3 (see main text for explanation).

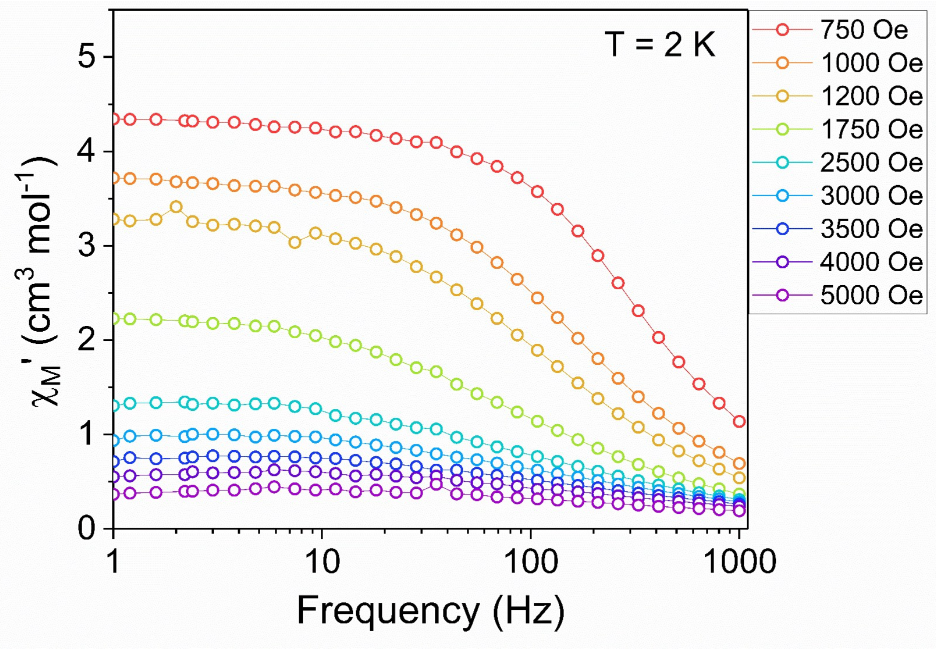

Figure S21. Frequency dependence of the in-phase component (χ') of the ac susceptibility for **1** at T = 2 K with a 2 Oe switching field and applied dc fields varying from 750 to 5000 Oe. Lines are a guide for the eye.

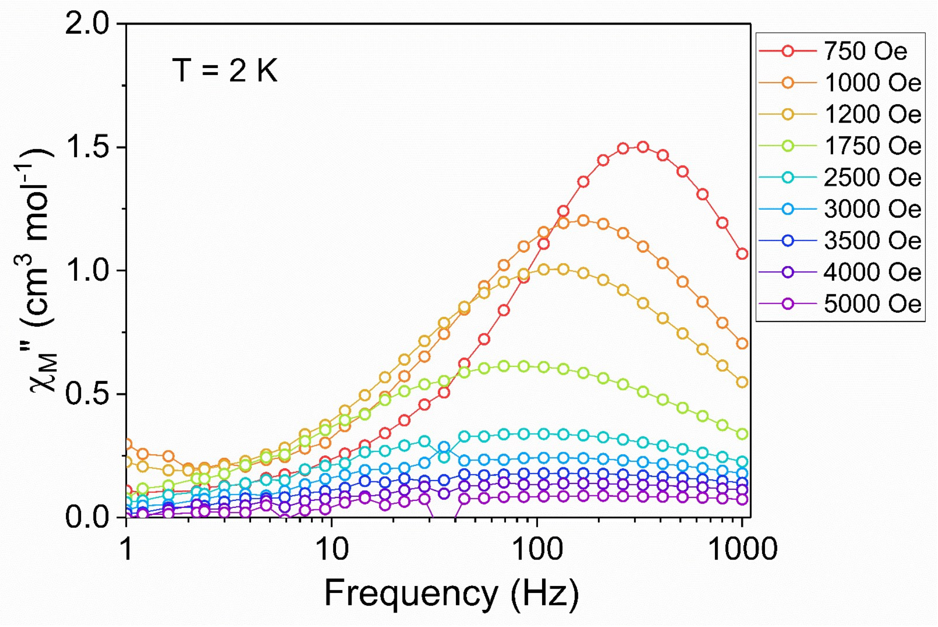

Figure S22. Frequency dependence of the out-of-phase component (χ") of the ac susceptibility for **1** at T = 2 K with 2 Oe switching field and applied dc fields varying from 750 to 5000 Oe. Lines are a guide for the eye.

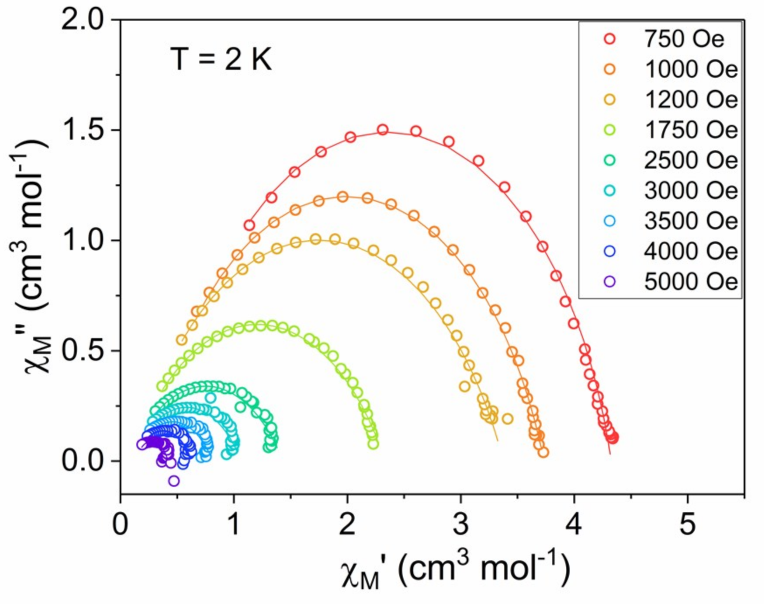

Figure S23. Cole-Cole plots for **1** at 2 K with various applied dc fields. Open circles are experimental data, solid lines are fits to the generalized Debye equation.

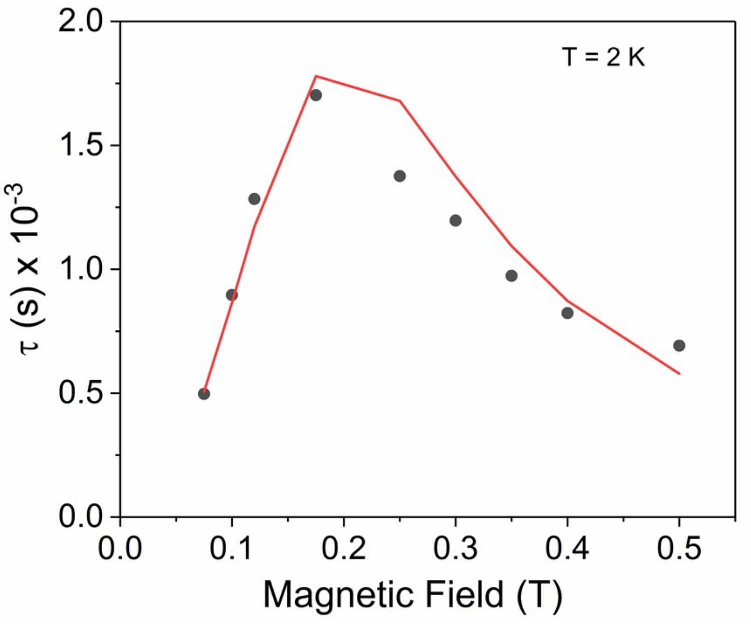

Figure S24. Field dependence of the relaxation times (τ) in **1**. Black circles are experimental data points, red line represents the fit to eqn 3 (see main text for explanation).

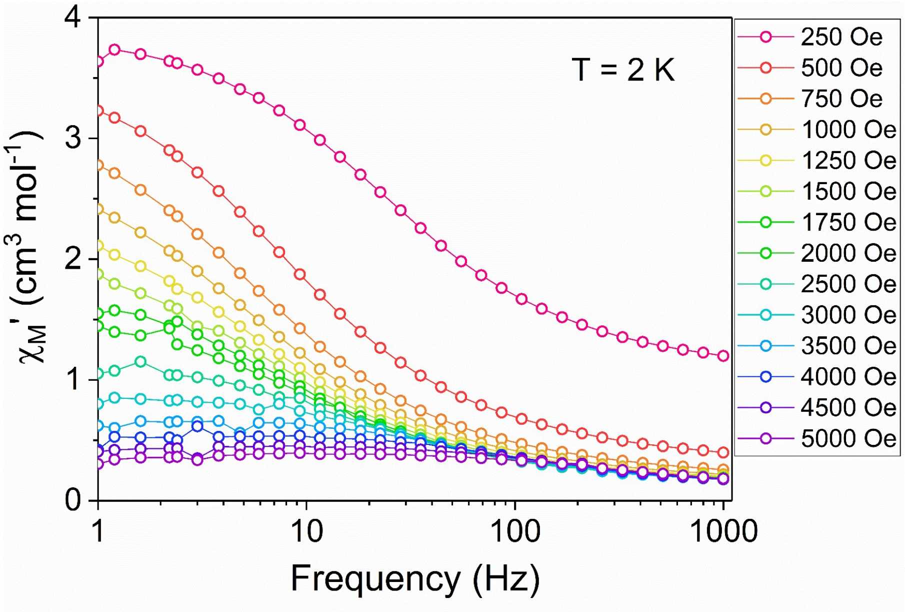

Figure S25. Frequency dependence of the in-phase component (χ') of the ac susceptibility for **[2]**$^-$ at 2 K with a 2 Oe switching field and applied dc fields varying from 250 to 5000 Oe. Lines are a guide for the eye.

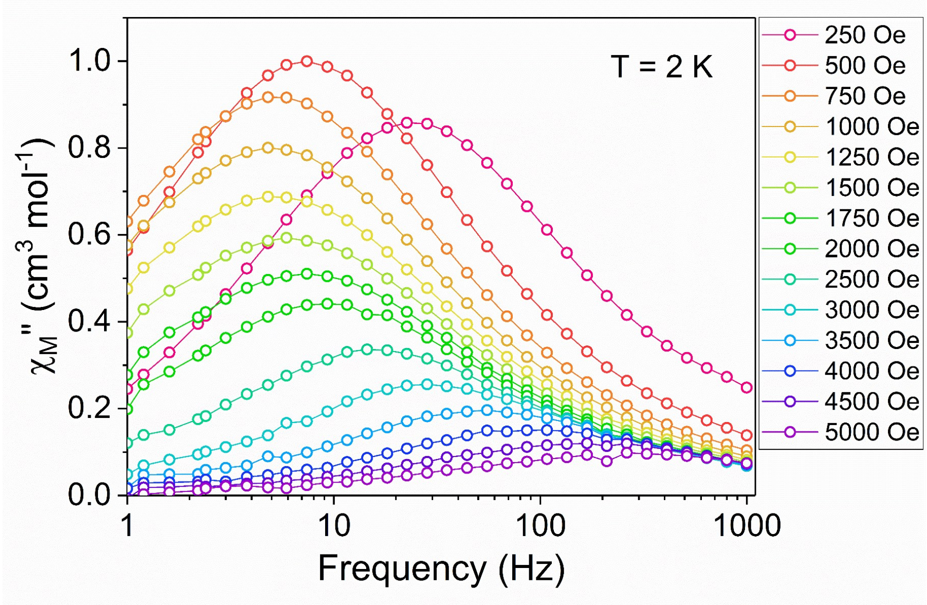

Figure S26. Frequency dependence of the out-of-phase component (χ") of the ac susceptibility for **[2]**$^-$ at 2 K with 2 Oe switching field and applied dc fields varying from 250 to 5000 Oe. Lines are a guide for the eye.

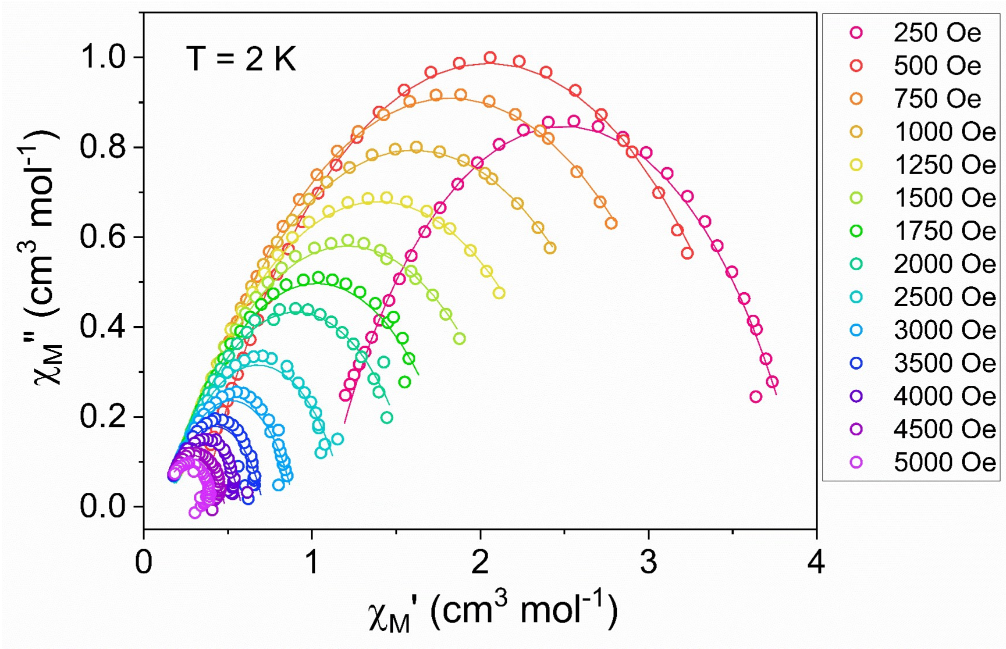

Figure S27. Cole-Cole plots for **[2]**⁻ at 2 K with various applied dc fields. Open circles are experimental data, solid lines are fits to the generalized Debye equation.

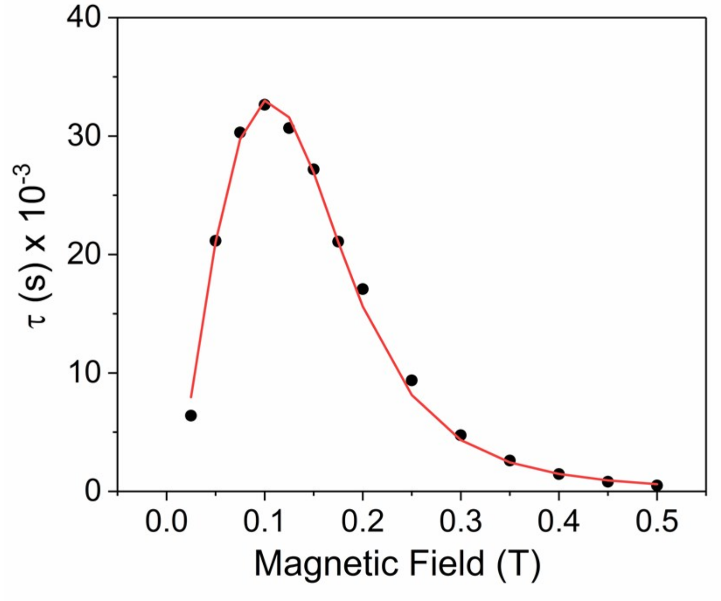

Figure S28. Field dependence of the relaxation times (τ) in **[2]**⁻. Black circles are experimental data points, red line represents the fit to eqn 3 (see main text for explanation).

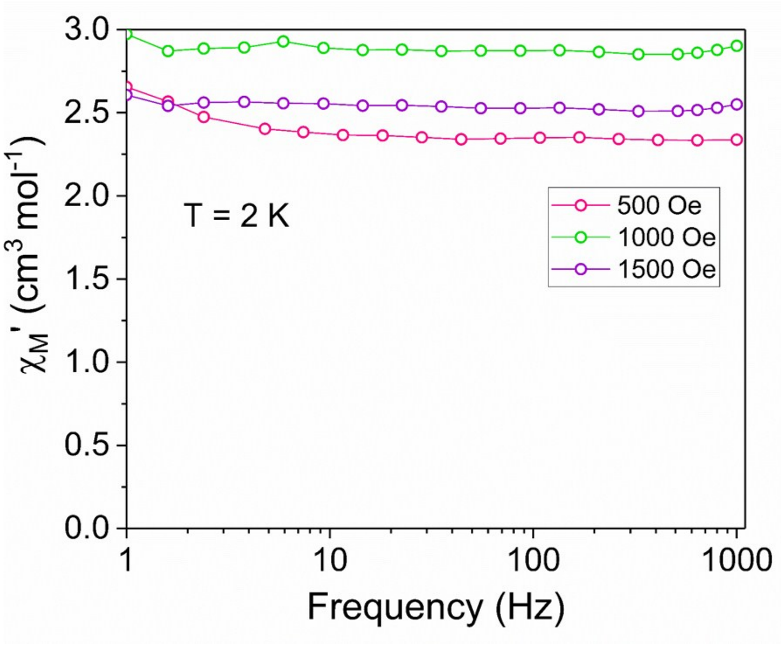

Figure S29. Frequency dependence of the in-phase component (χ’) of the ac susceptibility for **2** at 2 K with a 2 Oe switching field and applied dc fields varying from 500 to 1500 Oe. Lines are a guide for the eye.

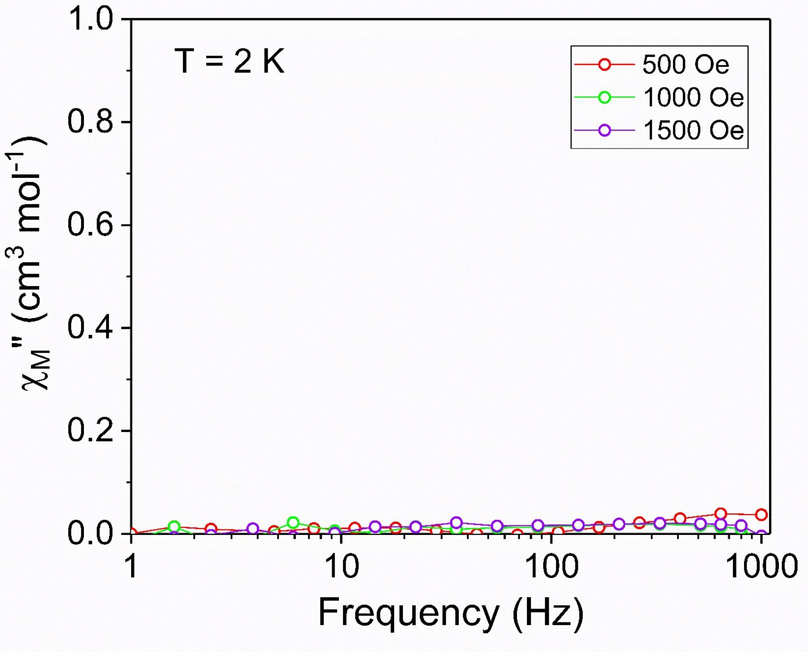

Figure S30. Frequency dependence of the out-of-phase component (χ”) of the ac susceptibility for **2** at 2 K with 2 Oe switching field and applied dc fields varying from 500 to 1500 Oe. Lines are a guide for the eye.

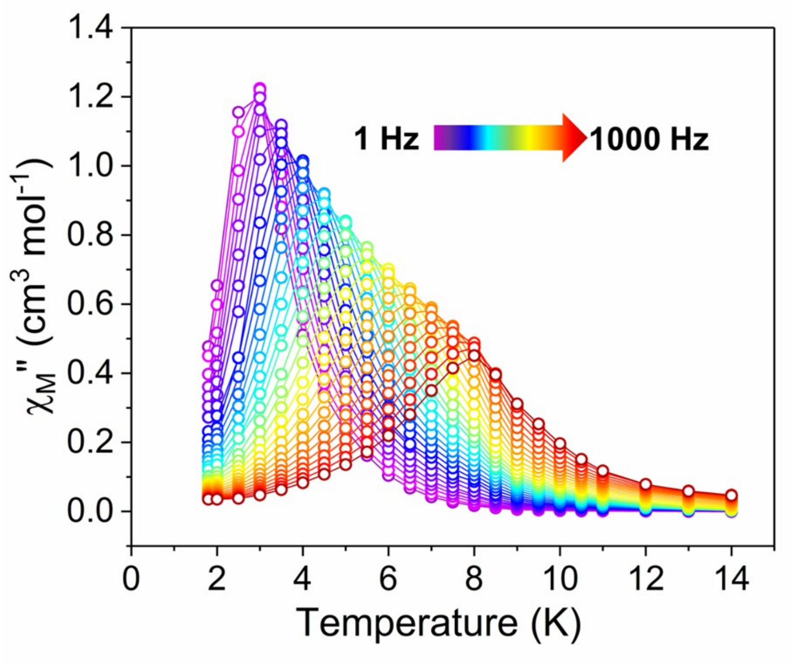

Figure S31. Temperature dependence of the out-of-phase component (χ”) of the ac susceptibility for **[1]**⁻ with a 2 Oe switching field and a 1000 Oe applied dc field (H_{dc} = 1000 Oe). Lines are a guide for the eye.

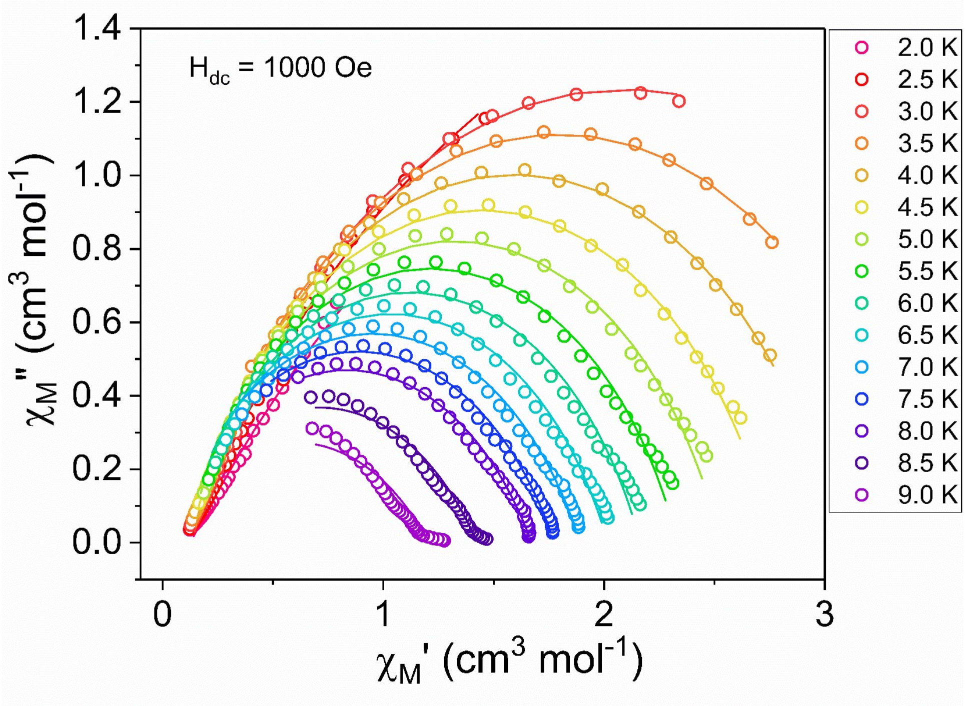

Figure S32. Cole-Cole plots for **[1]**⁻, with an applied dc field of 1000 Oe (H_{dc} = 1000 Oe). Open circles are experimental data points, solid lines are fits to the generalized Debye equation.

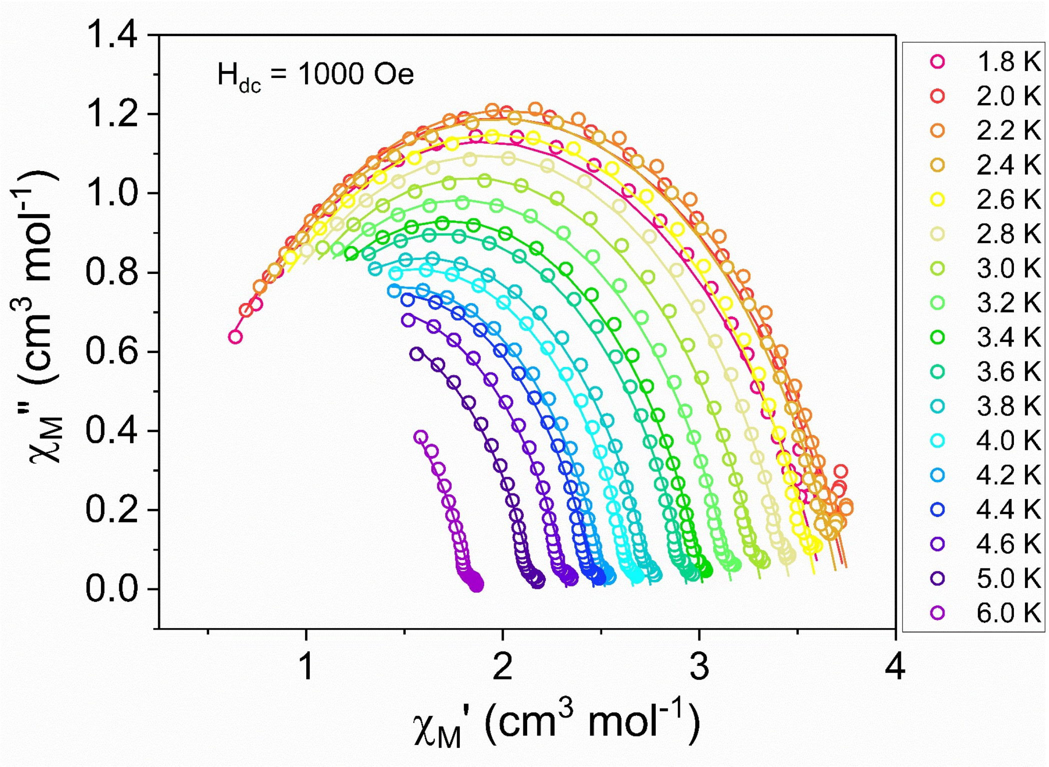

Figure S33. Cole-Cole plots for **1**, with an applied dc field of 1000 Oe (H_{dc} = 1000 Oe). Open circles are experimental data points, solid lines are fits to the generalized Debye equation.

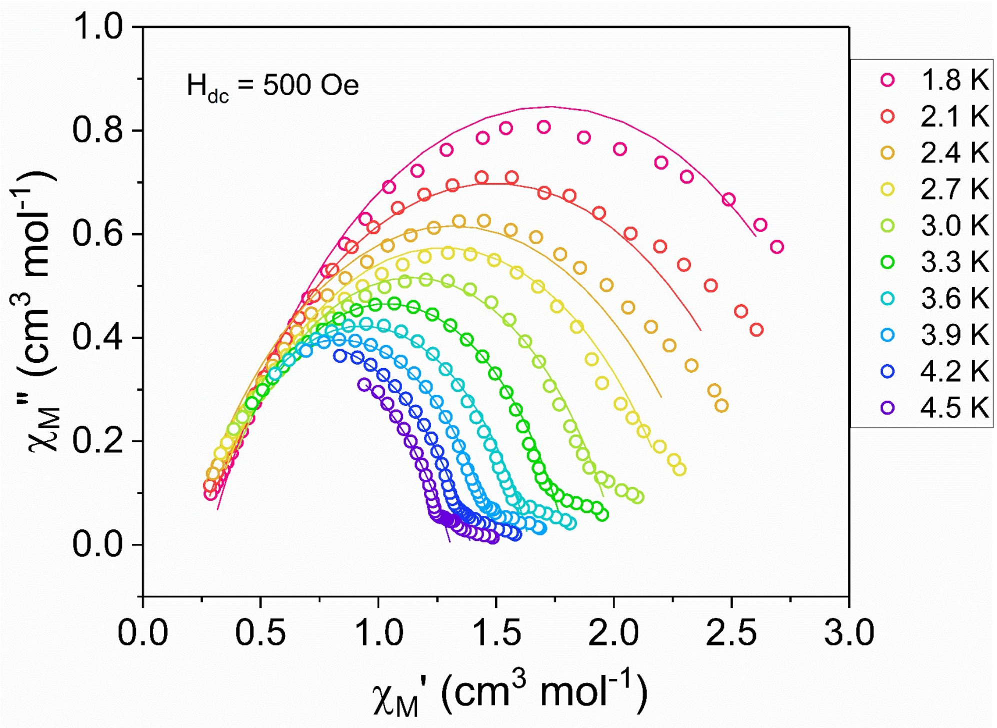

Figure 34. Cole-Cole plots for **[2]**$^-$, with an applied dc field of 500 Oe (H_{dc} = 500 Oe). Open circles are experimental data points, solid lines are fits to the generalized Debye equation.

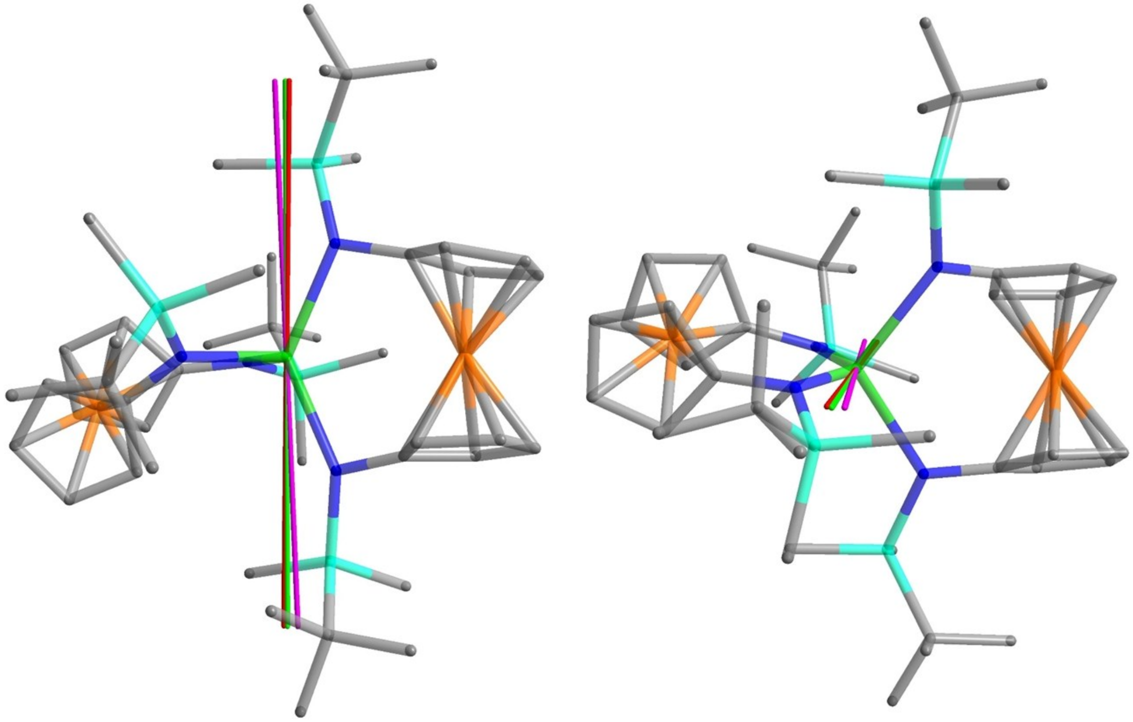

Figure S35. Predicted orientations of the magnetic anisotropy axes in **1** under three scenarios using MAGELLAN (Chilton *et al*): (1) red axis: assigning both Fe-Cp2 units as charge neutral (Fe^{2+}), (2) green axis: assigning +1 charge to the Fe centre that is closer to the Dy^{3+} ion, and (3) magenta axis: assigning the +1 charge to the Fe centre that is further from the Dy^{3+} ion. (ref. N.F. Chilton, D. Collison, E. J. L. McInnes, R. E. P. Winpenny and A. Soncini, *Nat. Commun.*, 2013, **4**, 1-7)

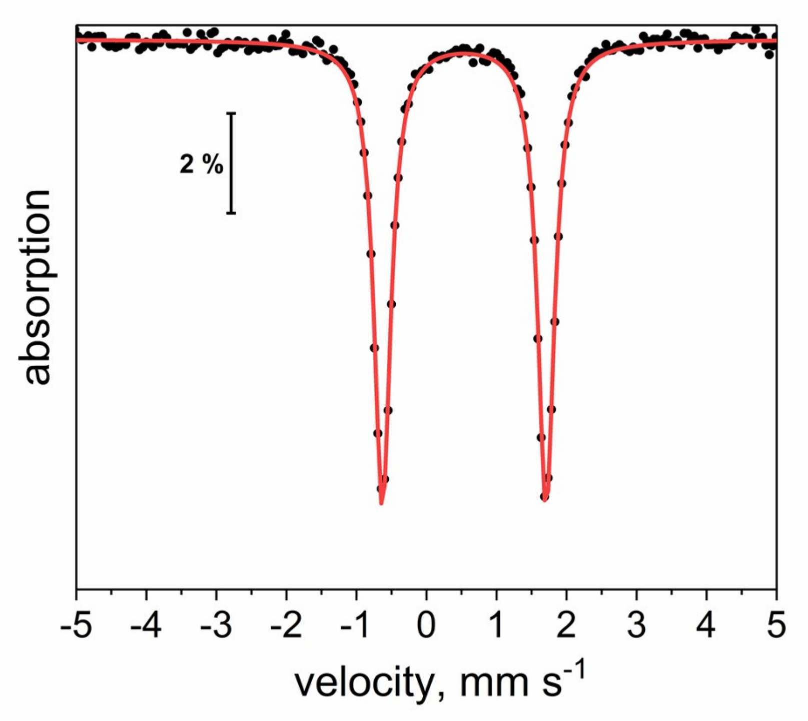

Figure S36. [57]Fe Mössbauer spectrum of **[1]**$^-$ at10 K with no external field. Isomer shift (δ) = 0.54 mm s^{-1}, quadrupole splitting (ΔE_Q = 2.34 mm s^{-1}).

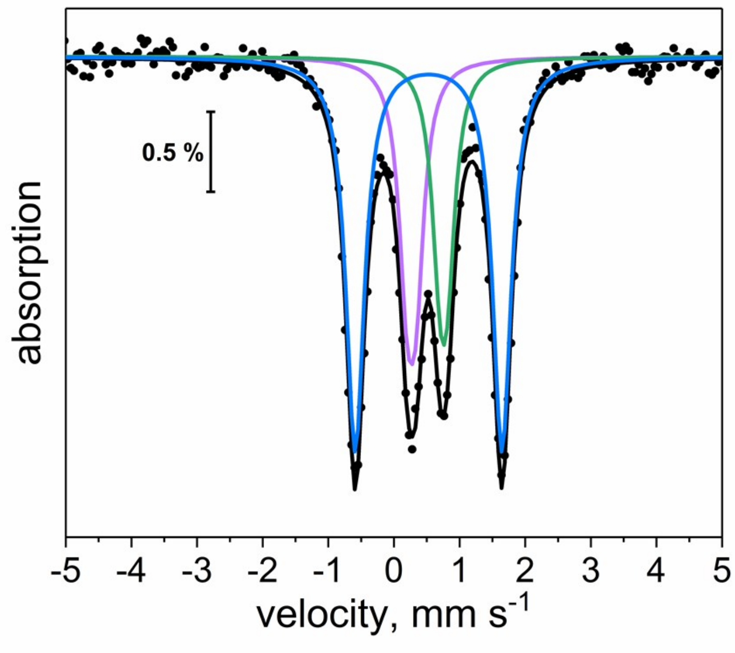

Figure S37. [57]Fe Mössbauer spectrum of **1**, at 5 K. Black dots are experimental points. Black line is overall three-site fit. Blue, green and purple lines are the individual sub-spectra for the three-site fit.

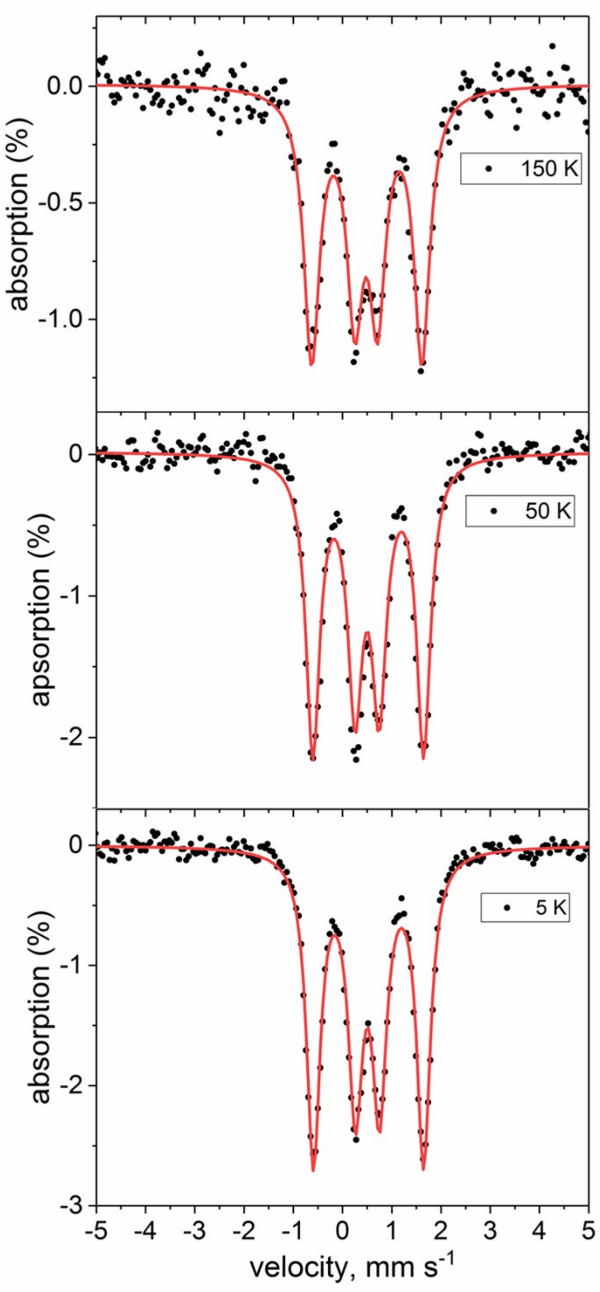

Figure S38. ^{57}Fe Mössbauer spectrum of **1** at 5 K, 50 K and 150 K. Black dots are experimental data points. Red lines are the overall two-site fits.

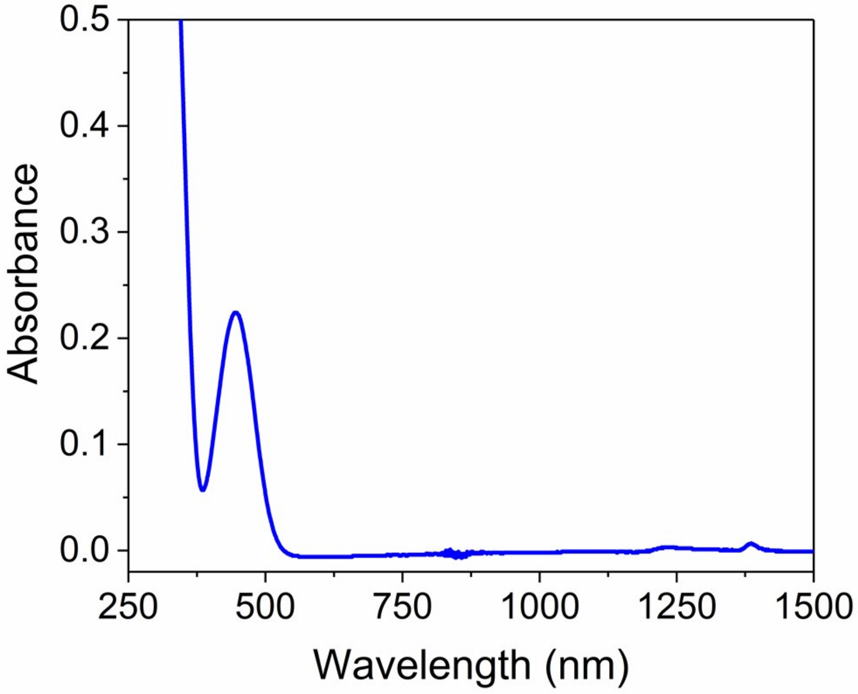

Figure S39. UV-vis-NIR spectrum of **[1]**$^{-}$ in thf.

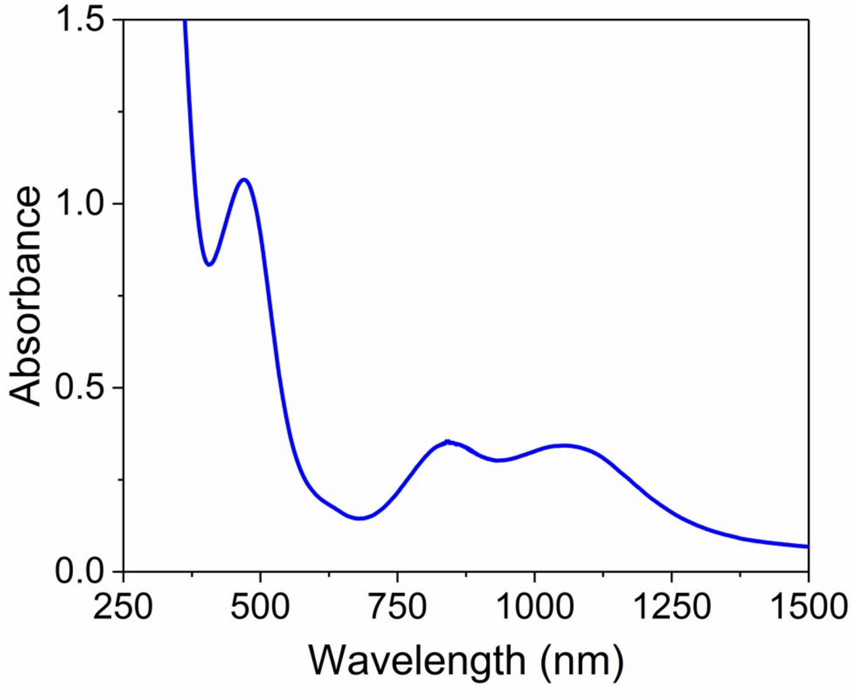

Figure S40. UV-vis-NIR spectrum of **1** in thf.

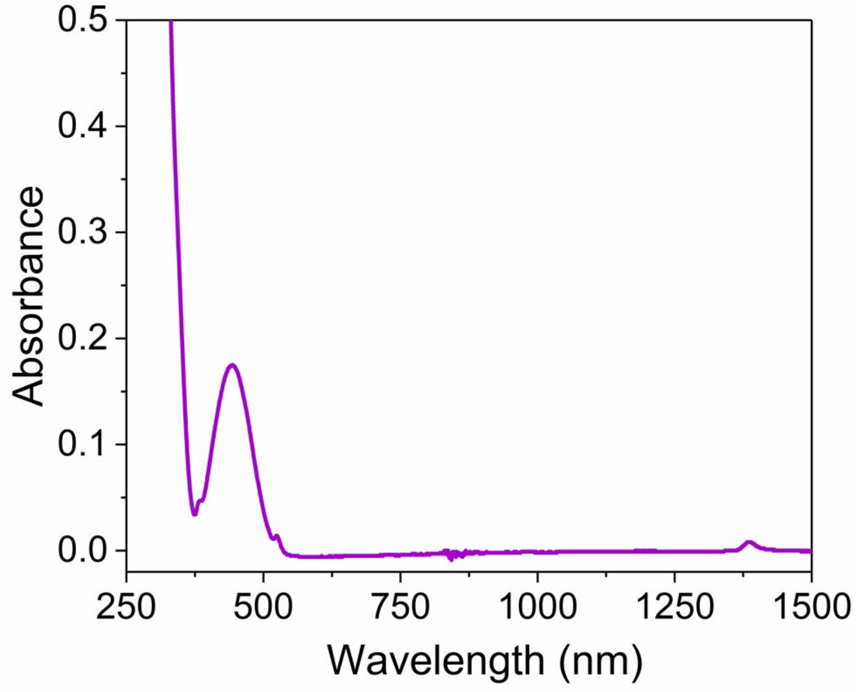

Figure S41. UV-vis-NIR spectrum of **[2]**$^-$ in thf.

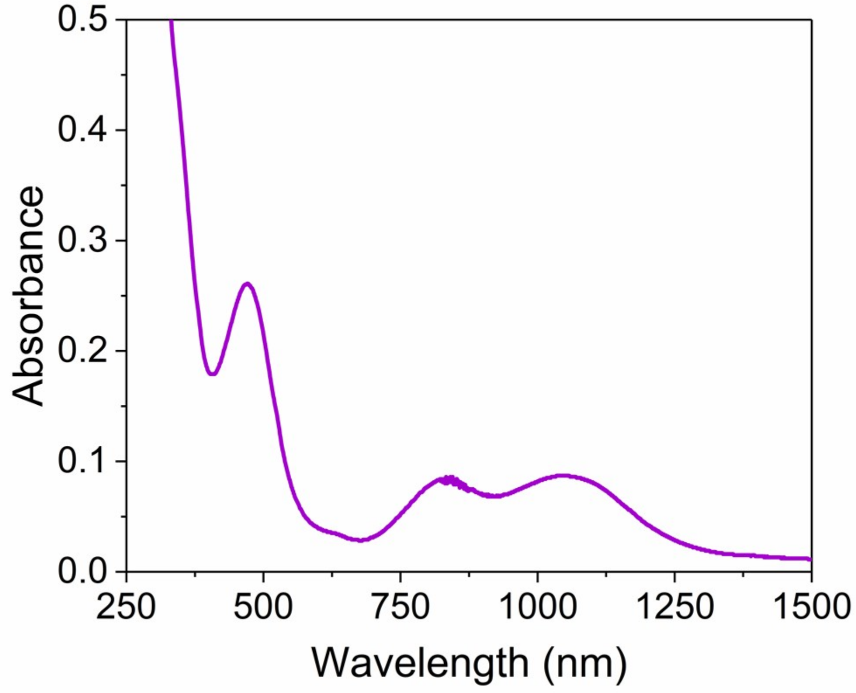

Figure S42. UV-vis-NIR spectrum of **2** in thf.
